# Extensive Microbial and Functional Diversity within the Chicken Cecal Microbiome

**DOI:** 10.1371/journal.pone.0091941

**Published:** 2014-03-21

**Authors:** Martin J. Sergeant, Chrystala Constantinidou, Tristan A. Cogan, Michael R. Bedford, Charles W. Penn, Mark J. Pallen

**Affiliations:** 1 Division of Microbiology and Infection, Warwick Medical School, University of Warwick, Coventry, United Kingdom; 2 School of Clinical Veterinary Science, Bristol, United Kingdom; 3 AB Vista Feed Ingredients, Marlborough, United Kingdom; 4 Institute of Microbiology and Infection, University of Birmingham, Birmingham, United Kingdom; Hospital for Sick Children, Canada

## Abstract

Chickens are major source of food and protein worldwide. Feed conversion and the health of chickens relies on the largely unexplored complex microbial community that inhabits the chicken gut, including the ceca. We have carried out deep microbial community profiling of the microbiota in twenty cecal samples via 16S rRNA gene sequences and an in-depth metagenomics analysis of a single cecal microbiota. We recovered 699 phylotypes, over half of which appear to represent previously unknown species. We obtained 648,251 environmental gene tags (EGTs), the majority of which represent new species. These were binned into over two-dozen draft genomes, which included *Campylobacter jejuni* and *Helicobacter pullorum*. We found numerous polysaccharide- and oligosaccharide-degrading enzymes encoding within the metagenome, some of which appeared to be part of polysaccharide utilization systems with genetic evidence for the co-ordination of polysaccharide degradation with sugar transport and utilization. The cecal metagenome encodes several fermentation pathways leading to the production of short-chain fatty acids, including some with novel features. We found a dozen uptake hydrogenases encoded in the metagenome and speculate that these provide major hydrogen sinks within this microbial community and might explain the high abundance of several genera within this microbiome, including *Campylobacter*, *Helicobacter* and *Megamonas*.

## Introduction

The domestic chicken occupies a special place in science and society. It is the most common domesticated animal, the most important food production animal and the most abundant and widely distributed bird in the world, scattered across islands and continents from the Arctic to the Falklands. Chickens provide a case study in domestication, a process began >7,000 years ago in South-East Asia [1]. Chickens have also played a key role in the history of science, from Aristotle's description of a chick embryo [2] to Darwin's account of their domestication [3]. The chicken has been adopted as a laboratory organism for research in genetics, embryology, development, immunology, virology and cancer [4]. The completion of the chicken genome sequence was a landmark in basic and applied biology [5]. However, the nuclear and mitochondrial genomes of the chicken represent just a small part of the gene pool associated with this bird.

Chickens, like other birds, have proportionally smaller intestines and shorter transit digestion times than mammals, but do not appear to any less efficient at digestion than their mammalian counterparts [6]. This may be explained, in part, by the fact that the chicken gastrointestinal tract is home to a complex microbial community, the chicken gut microbiota, which underpins the links between diet and health in this bird. The healthy chicken gut microbiota plays important roles in assimilating nutrients from food, in producing vitamins and essential amino acids and in preventing harmful pathogens from gaining a foothold [7]. The chicken gut microbiota also acts as source of human infections and as a reservoir of antibiotic-resistance determinants [8]. An optimal gut microbiota can increase agricultural productivity, as evidenced by the ability of antibiotics to promote growth in chicks [9]. This microbiota is also home to a rich collection of genes, the chicken gut microbiome, likely to include many sequences of scientific interest and biotechnological potential.

The chicken gastrointestinal tract consists of a crop, where food can be stored, followed by a proventriculous or true stomach which leads to the muscular gizzard, where digesta are ground, before entering the small and finally large intestine. The most densely populated microbial community within the chicken gut is found in the ceca, a pair of blind-ended sacs that open off the large intestine [10]. At the entrance to each cecum, long intertwining villi sieve out coarse particulate material, allowing only soluble or fine particulate digesta to enter the lumen. Whereas the transit time through the upper intestine is only 2.5 hours, digesta dwell in the ceca for 12–20 hours, potentially *a*llowing a longer time for digestion and absorption of nutrients. Various roles have been assigned to the cecal microbiota in wild birds [10] but its function in the modern commercial broiler is unclear and, *i*n some experiments, the caeca have been removed without impairing the chicken's development [10]. However, this microbiota has been implicated in many processes; nitrogen recycling by breakdown of uric acid [11], supplying B vitamins to their hosts [12] and producing essential amino acids required by their host [13]. Particularly relevant to the intensive farming of chickens is the cecum's role in digestion of non-starch polysaccaharides NSPs [14], which are found in the grains used in commercial chicken feed. Some authors suggest as much as 10% of energy needs is recovered from a well-functioning cecum [14], [15], with chicken fed high fiber showing a dramatic increase in the size of this organ [16]. The fermentation of breakdown products of NSPs produces short chain volatile fatty acids (SCFA), which are absorbed across the mucosa and catabolised by the host [6]. Such SCFA not only contribute to the nutrition of the chicken, they also lower pH which can inhibit acid-sensitive pathogens and improve mineral absorption [7]. In particular, butyrate has been shown to reduce *Salmonella* infection [17] and it is also able to stimulate epithelial cell growth [18]. Molecular hydrogen produced during the fermentation process [16] if allowed to accumulate can lead to inhibition of this process. Therefore, for efficient fermentation this gas needs to be removed by hydrogen-consuming bacteria [19]. These interactions are poorly understood and therefore the current study used metagenomics to identify pathways in the chicken ceca microbiome for NSP breakdown, SCFA production and hydrogen consumption.

Previous culture-based studies have established that, as in other vertebrates, the phylums *Bacteroidetes* and *Firmicutes* predominated in the chicken cecal microbiota [20]–[22]. Attempts at culture-independent phylogenetic profiling using 16S rRNA gene sequences have yielded insights into the composition of the chicken cecal microbiome [23]–[27] and more recent studies have shown the domination by *Fimicutes*, especially of the *Clostridiales* order [28]. Similarly, studies using metagenomics, the whole-scale sequencing of DNA extracted from a microbial community without target-specific amplification or culture, have provided glimpses into the function of this complex microbial community [29], [30]. However, most previous studies have examined pooled samples, thus ignoring variation in individual chickens, and have not achieved sufficient depth of coverage to provide a global picture of the taxonomic diversity and functional repertoire of the whole microbiome. This study aimed to address these points and analyzed cecal contents from 10 broiler chickens from the same flock on a commercial farm to an unprecedented depth, and on one sample produced the largest metagenomic analysis to date in order to illuminate the function of the cecal microbiota in chickens.

## Experimental Procedures

### Ethics statement

In accordance with the United Kingdom Animal (Scientific Procedures) Act of 1986, this study did not require a Home Office project license because no regulated procedures were carried out. Chickens were humanely killed at a designated establishment by cervical dislocation, which is an appropriate method under Schedule 1 of the Act.

### Samples collection, DNA extraction and PCR amplification

Cecal samples were collected from Ross broilers at 42 days, housed indoors under standard commercial conditions. They had been fed on a wheat based diet with 5% maize which contained ionophores but no antibiotics. The feed conversion ratio was standard for the breed and there was no feed withdrawal period before the birds were sacrificed. The cecal contents were a typical brown colour and like toothpaste in constituency. Birds were euthanized by cervical dislocation and the ceca removed and transported to the laboratory on ice. The cecal surface was sterilized with 70% ethanol, a longitudinal incision was made with a scalpel and the edges pulled back. Contents were removed into a sterile tube and flash frozen in liquid nitrogen and stored at −70°C. DNA was extracted and PCR amplification of 16S rRNA sequences was performed as described previously [31] using primers F20 and R19 flanking the variable regions V1–V3 and an annealing temperature of 55°C. Sequencing of amplicons was carried out on the Roche FLX Titanium instrument as described previously [31].

### Phylogenetic analysis of 16S rRNA gene sequences

Reads were truncated where flow signals were less than 0.7 and all sequences were trimmed to 600 flows (around 350 bp in length). Clustering of the reads into Operation Taxonomic Units (OTUs) which share 97% identity was achieved using Uparse [32]. Other methods were also attempted (Text S1 and Figure S5). The following modifications to the standard Uparse pipeline were carried out. Pre-clustering (-cluster_smallmem) was included, which clustered sequences exhibiting two or less nucleotide difference. In addition, an extra chimera checking step (-uchime_ref) against the default reference database was performed. The resulting sequences were taken as defining putative operational taxonomic unites (OTUs) or phylotypes. OTU sequences were assigned to a taxonomic lineage using QIIME (assign_taxonomy.py) [33]. In addition, all OTU sequences were BLAST-searched against the NCBI nr database.

The resultant sequences, frequencies and taxonomic assignments were formatted into an OTU table that was compatible with the QIIME pipeline [33]. QIIME was used to calculate Simpsons' index and construct rarefaction curves. Dendograms depicting the similarity of bacterial communities were constructed via the jackknifed_beta_diversity script from QIIME, exploiting the Bray Curtis method to compute a similarity matrix and then the un-weighted pair-group method with arithmetic mean (UGPMA) to cluster the results. Jacknifed support was included to account for the different sampling depths. A hundred rarefied tables were generated at sample size that corresponded to the number of reads in the smallest sample to produce bootstrap values.

### Metagenome sequencing and analysis

DNA extracted from cecum 1A was used for metagenome sequencing. Library preparation and sequencing on one channel of the Illumina MiSeq 2000 were performed according to the manufacturer's instructions by Source BioScience, Nottingham UK.

Preliminary analysis of the data involved blasting 260 000 reads against the NCBI nr database and obtaining taxonomic from the output using MEGAN(45). A comprehensive analysis was then carried out and is summarized in Figure S4a. Initially, the reads were filtered and trimmed with CLC workbench (CLC bio, Denmark) using the default settings and reads below 60 bases were removed. The 81,772,788 reads that remained were assembled into contigs (environmental gene tags) using CLC work bench under the default settings. Coding sequences (CDSs) were predicted using MetaGene [34] and translated into protein sequences which were searched against the NCBI NR database using BLASTP. BLAST results were fed into MEGAN [35], which provided KEGG and SEED annotations as well as assigning a taxonomic origin using the software's LCA algorithm. Coverage of each environmental gene tag (EGT) was assessed by mapping reads to the EGT using BWA [36].

A taxonomic assignment was made for each EGT whenever >50% of the CDSs from the EGT had been assigned by MEGAN to the same taxon, otherwise the EGT's taxonomic assignment was scored as unknown. Functional domains were assigned to each predicted protein using Hidden Markov Models (HMMs). This was achieved using hmmsearch from HMMER [37] against the PFAM [38] and TIGRFAMS databases. Signal sequences were predicted using SignalP [39] and antibiotic resistance assigned using the ARDB package [40]. Known patterns were identified in the data using stand alone tool from the PROSITE web site [41]. All the information was stored in a MySQL database (Figure S4A) for easy retrieval and linking of the different threads of information.

### Binning of EGTs

Z scores for the 256 tetra-nucleotides in each EGT were calculated as described previously [42]. Pairwise comparisons of EGT Z scores were performed using the Pearson correlation coefficient and the results assembled into a matrix. The matrix was then used to cluster EGTs at various identity levels using the cluster.classic command from MOTHUR [43]. Initial seed clusters were obtained at 0.25 identity. Clusters were merged into putative genomes if they had similar depth-of-coverage and an identical taxonomic assignment at the family level. The validity of putative genomes was assessed by determining whether the house-keeping genes, *rpo*B, *rec*A, *gro*EL and *gyr*B were single copy. Taxonomic status confirmed by when bacterial ‘core’ genes from the human microbiome project (http://hmpdacc.org/tools_protocols/tools_protocols.php) were blasted against putative genomes with a cut off value of 1e^−10^. Completeness of putative genomes was assessed by comparing the size of the genome with that of the closest relative in the GenBank.

### Functional domain analysis

Most glucosyl Hyrdrolase (GH) domains were detected using PFAM HMMs as described previously [44]. To detect GH domains without an HMM, metagenomic protein sequences were blasted against a set of GH domains described previously [44] with an e-value cut off 10^−6^. Xylanase, licheninase and cellulase sequences were detected using predictions of enzyme activities from the SEED and KEGG annotations, combined with the appropriate GH domains as specified by the Cazy Website [45] (Table S6). Gene contexts were visualised in Artemis.

Fermentation to acetate was examined by looking for putative acetate kinase (TIGR0016) and phosphate acetyltransferase (TIGR00651) that were adjacent to each other in EGTs. To investigate fermentation to butyrate, 3-hydroxybuturyl-CoA dehydrogenase (BCD), models were constructed using HMM, based on 10 BCD proteins from divergent prokaryotes. Putative BCD genes were indentified using this model and were further verified by looking for an adjacent Electron Transfer Flavoprotein (PF01021). Putative butyryl-CoA:acetate Coa transferase genes was found by using the conserved sequence AFVDIAKAG [46]. The alternative pathway was identified by adjacent transbutyrylase (TIGR02706) and butyrate kinase (TIGR02707) genes. Fermentation to propionoate was investigated by searching for the methyl-Malonyl-CoA:pyruvate transcarboxylase gene using as a SEED/KEGG annotation of EC 2.1.3.1 and the epimerase gene was putatively identified using a SEED annotation of EC 5.1.99.1 and TIGR03081. Methylmalonyl decarboxylase genes were identified by a SEED/KEGG annotation of EC4.1.1.41 and TIGR01117.

Putative uptake hydrogenases were identified with the patterns [EGMQS]RXC[GR][IV]CXXX[HT]XXX[AGS]X(0,4)[VANQD] or [AFGIKLMV][HMR]XX[HR][AS][AFLY][DN]PC[FILMV]XC[AGS]XH [47]. The dissimilatory 5′ phophosulfate resuctase (APS) present in suphate reducers was putatively identified if genes with TIGR02064 and TIG02066 models were adjacent to each other. The AcetylCoa Synthase beta subunit responsible for the CO dehydrogenase reaction in acetogens was identified by the conserved sequences LCGAVSW and PMTSCGC [48]. A search for the methyl-coenzyme M reductase present in methanogens was performed by looking for adjacent genes containing TIGR03256 and TIG03257 domains.

## Results and Discussion

### Microbial diversity in the chicken cecum

**Figure 1:**
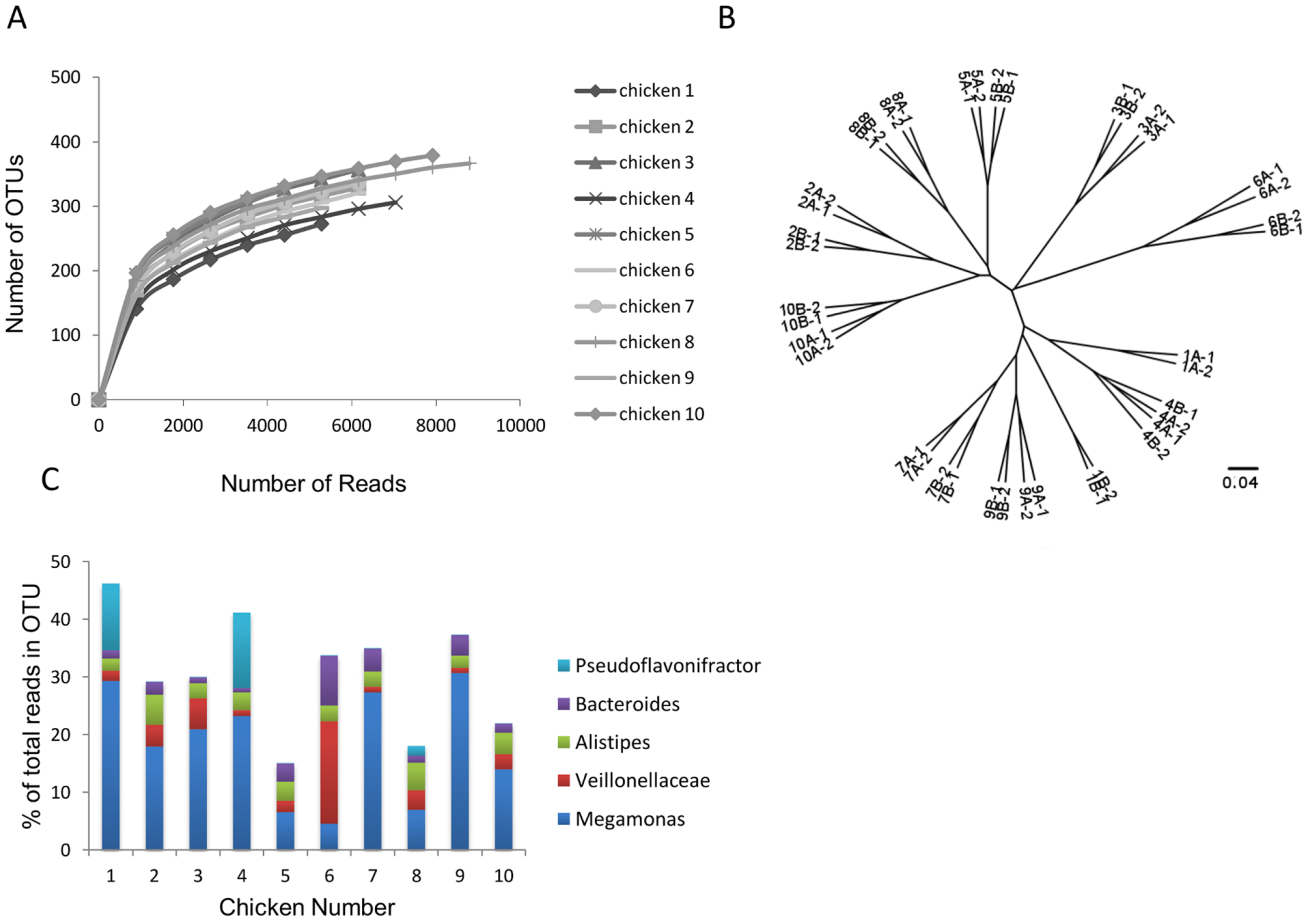
Summary of 16S analysis. (A) Rarefaction curves of the OTUs clustered at 97% sequence identity for each chicken, based on the averages of the four replicates per bird. (B) Hierarchical clustering visualizing similarities among cecal samples. The number in each label indicates each individual chicken and the letter each individual ceca (either A or B). Technical replicates of each ceca were carried out. (C) Bar chart showing the proportion of reads assigned to the top 5 most abundant OTUs in each chicken.

From the ten chickens, we recovered 414,070 16S sequences, which were classified into 699 phylotypes using a 97% sequence identity threshold. Rarefaction curves suggest that sampling has reached saturation and that the number of phylotypes in individual chickens ranges from ∼200 to ∼350 (Figure 1A). Sequences from 232 phylotypes showed less than 97% identity to any sequence in GenBank (Data Set S1) and therefore potentially represent new species. The majority of these OTUs contained large numbers of reads and hence are unlikely to be due to sequencing or PCR errors (Figure S1).

Previous studies have shown large inter-chick variation of microbiota community structure with both T-RLFP [49] and quantitative PCR [50], so we were curious to determine how much variation occurs within the same region of the gut. We analysed biological replicates from each bird (two ceca) and technical replicates for each sample and found that samples from the same bird clustered together, with the exception of bird 1(Figure 1B). Furthermore, in all but one case (bird 4), individual ceca from the same chicken could be differentiated with technical replicates clustering together. Simpson's diversity index varied between ceca and birds, with the highest value (i.e. least evenness) in bird 1 (Table S1), which was dominated by *Megamonas* and *Alistipes* (Figure 1C).

Curiously, in our samples, *Lachnospriceae*, *Ruminococcaceae* and *Bacteroides* appear to be less abundant than might be expected from previous studies (Figure S2). This can largely be explained by the unprecedented numerical dominance of the class *Negativicutes* (over 20% of all sequences) and in particular, by the high prevalence of a single novel species of *Megamonas*, which accounts for nearly a third of the reads in one of the birds, chicken 9 (Figure 1C).

### The chicken cecal metagenome

We obtained a hundred and twenty million 110-base reads from the contents of a single cecal sample (cecum 1A). A taxonomic analysis of a subset of reads (Table S2) showed that, of the reads that could be assigned to a taxon, >98% were bacterial, with <1% assigned to the chicken and fewer than one in a thousand assigned to archaea, other eukaryotes (e.g. the cereal fungus *Magnaporthe orazae* and the alga *Chlamydomonas reinharrdii*) or to viruses (bacteriophages and Fowl Adenovirus).

Reads were incorporated into a 337-Mbp assembly, with an N_50_ of 647 bp and consisting of 648,251 environmental gene tags (EGTs). From this assembly, we predicted 108,103 complete coding sequences (CDS) and 595,178 partial CDSs. Of the EGTs greater than 2 kb about 13% could be assigned to a known species (Figure S3A), suggesting that the majority of EGTs that we have recovered represent new species. Seven percent of EGTs could not even be assigned to a known domain (Figure S3A). The majority of these showed, on homology searching, just a few weak matches to phage genes. Since phages largely contain genes with unknown functions [51] these EGTs probably represent phages.

When the taxonomic distribution predicted from the 16S analysis is compared with that from the metagenomics data, there is a discrepancy in the ratio of *Firmicutes* to *Bacteroidetes* (Figure S3b). This is largely due to differences in the ratio of reads from the genus *Alistipes*, which belongs to the *Bacteroidetes*, to those from *Megamonas*, which belongs to the *Firmicutes*, (19.9 and 16.3% in the 16S data set; 32% of and 9% in the metagenome), probably reflecting differential amplification in the 16S PCR.

EGTs were binned according to Z-score, coverage and taxonomic assignment, allowing us to reconstruct two-dozen genomes larger than 1Mbp (Table 1). Only five genomes showed a ≥99% match within the rpoB gene to sequences in GenBank; suggesting most of the draft genomes we have recovered represent new species.

**Table 1:** Draft genome sequences recovered from the chicken cecal metagenome.

Draft Genome ID	# of contigs	Size (Mbp)	G+C %	Depth of Coverage	Taxonomic Assignment	Closest Genome	Similarity to closest genome^a^	Size of closest genome	Completeness by closest genome^b^	Completeness by core genes^c^
4	555	5.49	44	21	*Bacteroides*	*Bacteroides dorei 5_1_36/D4*	100%	5.53	99%	86%
1	761	3.30	50	23	*Enterobacteriaceae*	*Escherichia albertii TW07627 a*	100%	4.70	70%	24%
3	570	3.01	58	31	*Firmicutes*	*Clostridium cellulovorans 743B*	58%	5.26	57%	81%
29	91	2.80	49	67	*Clostridiales*	*Subdoligranulum sp. 4_3_54A2FAA*	90%	4.21	67%	93%
36	63	2.71	52	39	*Bacteroidales*	*Tannerella sp. 6_1_58FAA_CT1*	92%	3.84	70%	91%
2	636	2.59	53	19	*Lachnospiraceae* 3_1_57FAA_CT1	*Lachnospiraceae bacterium 3_1_57FAA_CT1*	94%	7.69	34%	27%
7	448	2.53	61	38	*Subdoligranulum variabile*	*Subdoligranulum variabile DSM 15176*	88%	3.24	78%	62%
34	77	2.51	42	254	*Veillonellaceae*	*Megamonas hypermegale ART12/1*	94%	2.56	98%	95%
45	37	2.44	62	942	*Alistipes*	*Alistipes sp. HGB5*	95%	3.46	71%	88%
16	218	2.31	58	27	*Bacteroidales*	*Alistipes indistinctus YIT 12060*	93%	3.09	75%	90%
5	521	2.30	56	14	*Victivallis vadensis*	*Victivallis vadensis ATCC BAA-548*	75%	4.58	50%	53%
38	56	2.25	54	79	*Clostridiales*	*Clostridium sp. MSTE9*	77%	3.51	64%	92%
14	257	2.20	61	39	*Bacteroidales*	*Alistipes sp. HGB5*	94%	3.46	64%	89%
6	508	1.93	63	40	*Ruminococcaceae bacterium D16*	*Lachnospiraceae bacterium 7_1_58FAA*	96%	3.21	60%	68%
23	129	1.81	56	57	*Bacteroidetes*	*Flavobacterium columnare ATCC 49512*	83%	3.16	57%	93%
30	89	1.80	57	41	*Dialister*	*Dialister succinatiphilus YIT 11850*	79%	2.46	73%	95%
44	38	1.73	29	47	*Campylobacter jejuni*	*Campylobacter jejuni 81-176*	99%	1.62	107%	89%
11	274	1.72	68	53	*Coriobacteriaceae*	*Atopobium parvulum DSM 20469*	86%	1.54	111%	88%
17	212	1.68	51	70	*Clostridiales*	*Ruminococcus torques L2-14*	94%	3.34	50%	89%
43	42	1.67	34	92	*Helicobacter pullorum*	*Helicobacter pullorum MIT 98-5489*	99%	1.92	87%	89%
8	364	1.50	45	13	*Bacteroides*	*Bacteroides thetaiotaomicron VPI-5482*	99%	6.29	24%	7%
12	297	1.48	64	94	*Bifidobacterium*	*Bifidobacterium gallicum DSM 20093*	93%	2.02	73%	79%
18	188	1.43	47	46	*Clostridiales*	*Clostridium leptum DSM 753*	74%	3.27	44%	86%
10	286	1.34	62	19	*Coriobacteriaceae*	*Coriobacterium glomerans PW2*	88%	2.12	63%	78%
15	222	1.30	50	15	*Clostridiales*	*Ruminococcus albus 8*	79%	3.69	35%	70%

a Closest genome was identified based on blastp of the translated *rpo*B gene from the draft genome against the ncbi nr database ^b^ Completeness based on the sum size of total contigs compared to the size of the closest genome ^c^ Completeness based on the % of core genes present (see methods).

Antibiotic resistance genes were investigated using the ARDB database pipeline [40] and the results are shown in data S2. The most common predicted resistance was against tetracycline and bacitracin. In addition a vancomycin resistance operon in *Enterococcus* was detected.

### Polysaccharide-degrading enzymes

**Figure 2:**
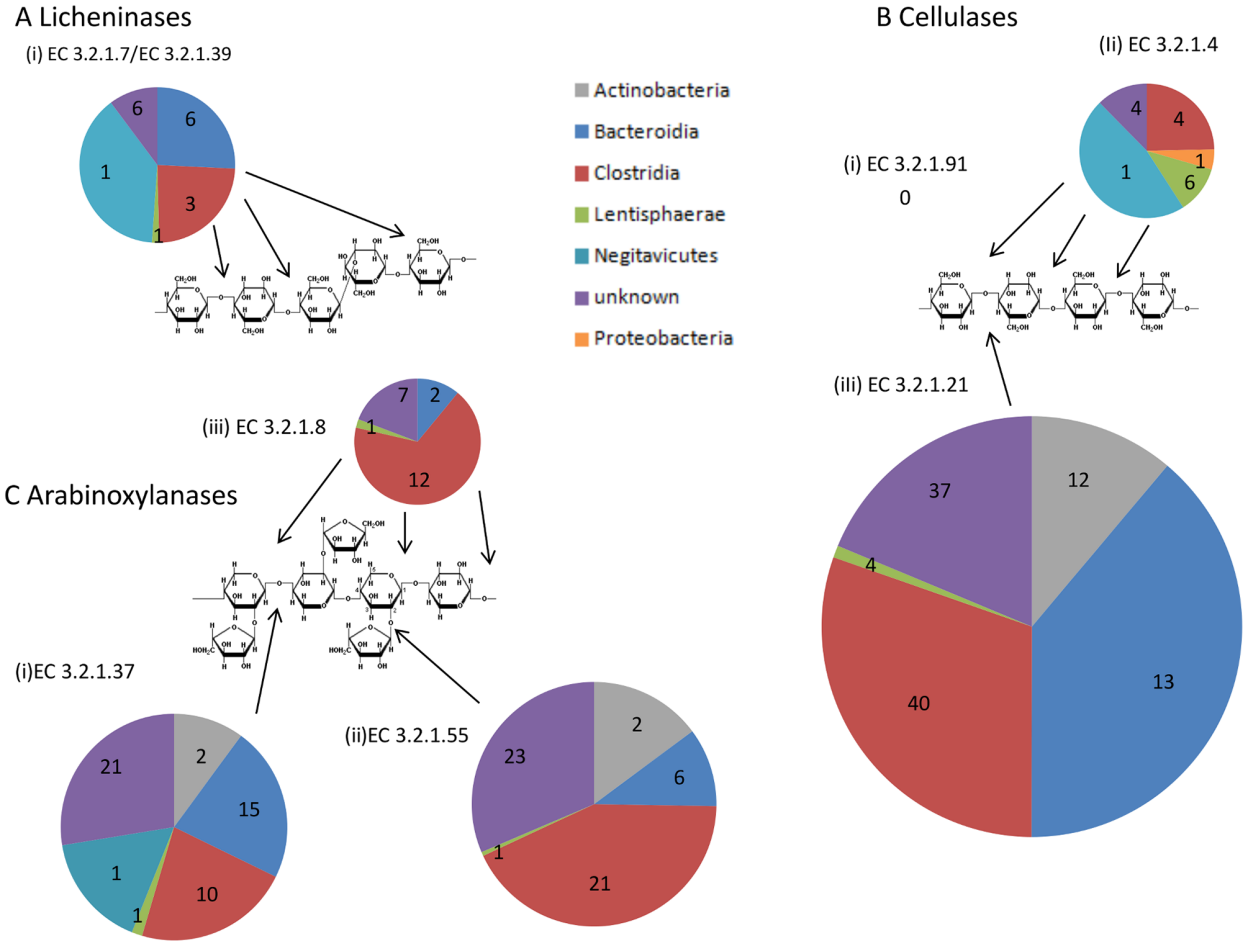
Polysaccharide-degrading enzymes in the chicken cecal metagenome. The figure shows each class of enzyme as judged by SEED/KEGG annotation and GH (see methods) for three types of NSP. The size of pie chart reflects abundance of the enzyme class; numbers indicate quantity of genes assigned to each bacterial Taxon at the class level.

The grain that forms the basis of most commercial chicken diets is rich in non-starch polysaccharides, with β-glucans, including cellulose, predominating in barley and oats and arabinoxylans abundant in wheat, maize and rye. In comparison to a set of other known metagenomes, the chicken cecal microbiome contains the largest proportion of sequences (1.5%) representing glycosyl-hydrolase (GH) domains (Table 2). Within this microbiome, we found sequences from over two hundred different non-starch polysaccharide-degrading enzymes (Figure 2).

**Table 2:** Number of genes containing GH domains in different metagenomes.

	Macropod^a^	Termite^b^	Bovine^c^	Panda^d^	chicken
Cellulases					
GH5	10	56	1451	3	151
GH6	0	0	0	0	0
GH7	0	0	1	0	0
GH9	0	9	795	0	14
GH44	0	6	?	0	41
GH45	0	4	115	0	0
GH48	0	0	0	0	0
Total	10(2)	74(11)	2365(9)	3(1)	206(2)
Endohemicellulases					
GH8	1	5	329	2	21
GH10	11	46	1025	2	60
GH11	0	14	165	0	0
GH12	0	0	0	0	0
GH26	5	15	369	0	18
GH28	2	6	472	1	93
GH53	9	12	?	0	21
Total	28(5)	98(14)	2360(9)	13(3)	213(2)
Debranching Enzymes					
GH51	12	18	?	5	84
GH54	0	0	?	0	0
GH62	0	0	1	0	0
GH67	5	10	120	2	12
GH78	25	0	1260	2	425
Total	42(8)	18(3)	1381(5)	9(2)	521(5)
Oligosaccharide-degrading enzymes					
GH1	61	22	253	101	263
GH2	24	23	1436	1	998
GH3	72	69	2844	18	1275
GH29	2	0	939	1	232
GH35	3	3	158	4	54
GH38	3	11	272	10	73
GH39	1	3	315	9	45
GH42	8	24	374	18	367
GH43	10	16	?	0	340
GH52	0	3	0	0	0
Total	184(33)	174(24)	6591(23)	162(36)	3647(38)
Sum	557	704	27755	448	9033
CDS	78896	82789	2547270	49844	595178
%GH	0.71	0.85	1.09	0.90	1.52

Data are grouped according to Allgier *et al* [68]. The numbers in parenthesis are relative to total number of GH containing genes. The data is take from ^a^wallaby [55] ^b^termite [44], ^c^panda [69] and ^d^bovine rumen [70].

In line with the expectation that less soluble, larger polymers are excluded from the cecum, we found that, in comparison to other microbial communities, the chicken cecal microbiome (along with the panda microbiome) harbored the lowest proportion of cellulases and endohemicellulases (4%), but the largest proportion of oligosaccharide degrading enzymes (38%) within GH-contiaing genes (Table 2). In particular, glucanases acting on oligosaccharides (E.C. 3.2.1.4 and E.C. 3.2.1.1) predominate over endoglucanases (EC 3.2.1.4 and E.C 3.2.1.8) acting on full-length polymers of cellulose and xylan (Figure 2). We found no evidence of any sequences representing the exo-acting cellobiohydralase activity (E.C. 3.2.1.91) associated with the degradation of crystalline cellulose [52]. We found a greater abundance of sequences involved in degradation of xylans than for degradation of β-glucans in the ceca, which contrasts with the relative dominance of β glucanase activity over xylanase in the small intestine [53].

Enzymes involved in xylan and cellobiose degradation are similarly distributed across three main bacterial classes, *Actinobacteria*, *Clostridia* and *Bacteroidia* (Figure 2). However, among endoglucanases, there appears to be a relative under-representation of the *Actinobacteria*, with greater contributions from the *Negativicutes* and *Lentisphaerae* (in particular the species *Victivallis vadensis*).

In order to reap the benefits of extracellular non-starch polysaccharides (NSP) degradation, bacteria exploit multi-protein cellulosomes, characterized by dockerin motifs, or isolated cellulases which use carbohydrate binding modules (CBM) to attach to the substrate [52], [54]. However, in our dataset, only 51 of 9498 GH domains were attached to binding modules and only two were attached to dockerin motifs (Table S3), again suggesting an absence or paucity of conventional cellulase activity.

**Figure 3:**
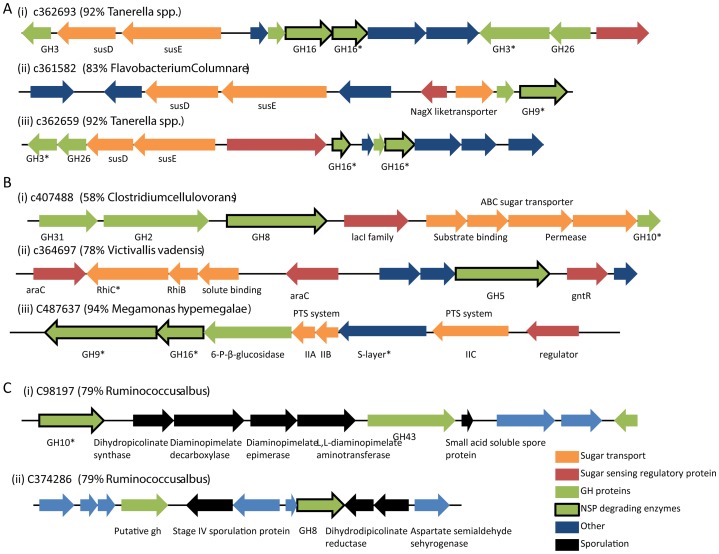
Gene clusters associated with polysaccharide degradation and utilization. NSP degrading genes were identified by SEED/KEGG annotation and GH domain (see methods). (A) Gene clusters encoding putative PUL systems from various *Bacteroidetes*. (B) Gene cluster encoding putative integrated polysaccharide degradation and utilization systems from various *Firmicutes*. (C) NSP degrading enzymes associated with sporulation genes * indicates predicted signal peptide.

Another complex enabling bacteria to exploit NSP degredation, which are present in *Bacteroides*, are polysaccharide utilization systems (PUL), which digest and import the products of polysaccharide degradation [55]. We found evidence of over 500 such systems in the *Bacteriodetes* from the chicken cecum, including representatives of a novel class of PUL recently identified in the Tammar Wallaby foregut that incorporates β-1,4 endoglucanases and β-1,4 endoxylanases [55] (Figure 3A).

We found additional genetic evidence of co-ordination of polysaccharide degradation with sugar transport and utilization (Figure 3B) – for example, in *Megamonas*, we found a gene cluster encoding secreted endo-glucanases, a cellobiose phosphotransferase system and a 6-phospho-beta-glucocidase, which together probably form an extracellular complex capable of degrading NSPs to cellobiose, which is then imported and cleaved to give D-glucose.

Some endoglucanases were not associated with regulatory genes or sugar transporters and therefore may not be involved in the exploitation of NSP for energy. For example, it has been suggested that some cellulase genes might be involved in biosynthesis of cellulose [56], as evidenced by their association in operons with cellulose synthesis genes. We found no evidence of biosynthetic cellulose activity in the chicken microbiome. However, we found two endoglucanase genes in a *Clostridiales* genome that were associated with sporulation genes (Figure 3C), which leads us to speculate that such endoglucanase genes may be implicated in breakdown of the cell wall or formation of the spore cortex.

### Production of short-chain volatile fatty acids

Short-chain volatile fatty acids (SCFAs), such as acetic acid, propionic acid and butyric acid, are produced by microbial fermentation of the sugars released from non-starch polysaccharides and are found in the chicken ceca [57]. SCFAs elicit several beneficial effects. Absorption of SCFAs across the gut mucosa contributes to the nutrition of the chicken (6). In addition, SCFAs can inhibit the growth of pathogens and improve mineral absorption [17].

**Figure 4:**
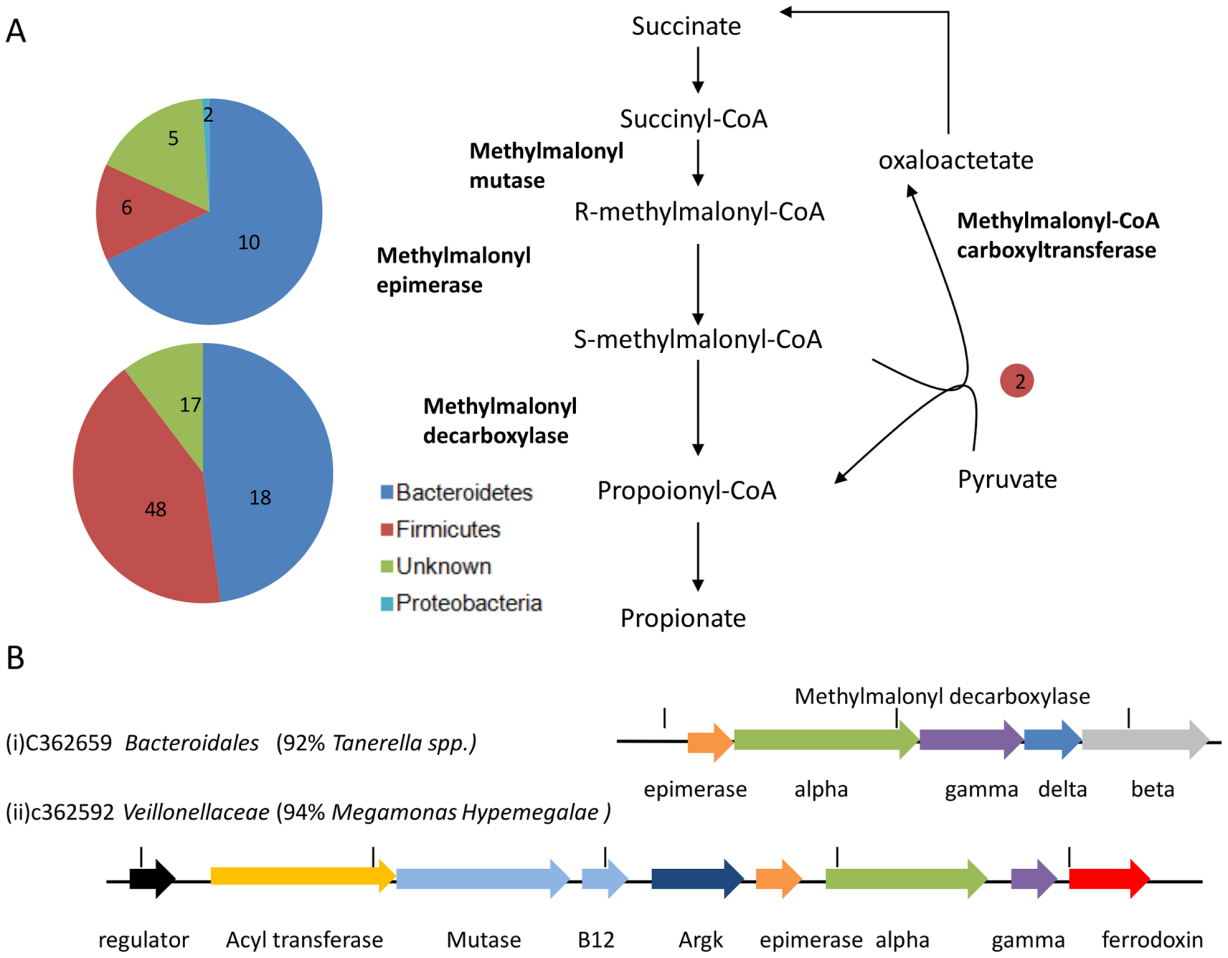
Pathways and gene clusters associated with propionoate production in the chicken cecal metagenome. (A) Pathways involved in propionoate production showing the putative genes identified coding for the enzymes involved. Size of pie chart reflects abundance of the gene class; numbers indicate quantity of genes assigned to each taxon. (B) Operon structure of two Methylmalonyl decarboxylase loci.

We found abundant evidence of acetate fermentation in the chicken cecal metagenome, with over 30 acetate kinase/phosphotransferase sequences (Table S4). One of pathways to propionate fermentation, the Wood-Werkman cycle, is poorly represented, with two methylmalonyl-CoA carboxytransferase genes found at low coverage (Figure 4A). However, an alternative propionate fermentation pathway, which exploits methylmalonyl-CoA decarboxylase and methylmalonyl-CoA epimerase appears to be well represented in this microbial community, with many genes encoding these enzymes (Figure 4A). As expected, we found that gene clusters from the *Bacteroidetes* that encode the alpha subunit also encode the beta, gamma and delta subunits of methylmalonyl-CoA decarboxylase together with methylmalonyl epimerase (Figure 4B). However, similar gene clusters from *Megamonas* and *Dialister* appear to lack genes for the beta subunit, which couples decarboxylation to export of Na^+^ across the membrane, and the delta subunit, which holds the complex together [58]. Instead these clusters encode a ferredoxin gene, which, we speculate, may be involved in coupling decarboxylation to electron transport in a novel propionate fermentation pathway.

The first committed step in butyrate production is the conversion of crotonoyl-CoA to butyryl-CoA by 3-hydroxybutyryl-CoA dehydrogenase (BCD) [59]. Assignment of this enzyme to KEGG or SEED categories is hampered by similarities of this enzyme to acyl-CoA dehydrogenases involved in lipid metabolism. In butyrate-producing bacteria the BCD gene is found in an operon adjacent to electron transferring flavoproteins (ETF). The BCD:ETF complex conserves the energy of the reaction, which is believed to be used to generate proton-motive force [60].

**Figure 5:**
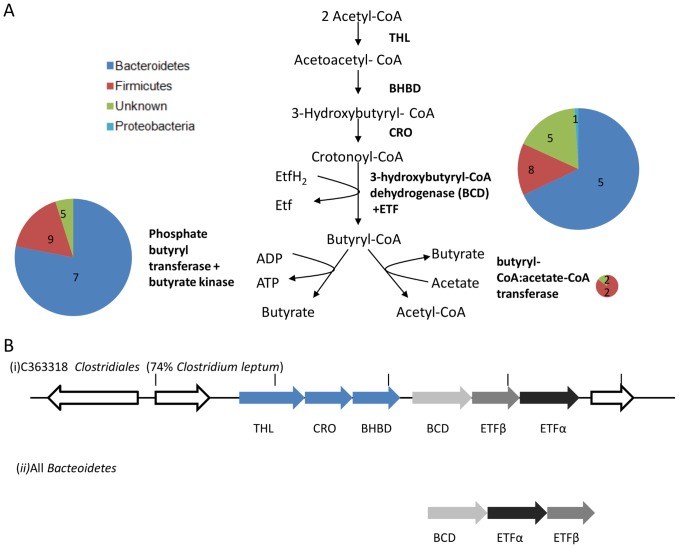
Pathways and gene clusters associated with butyrate production in the chicken cecal metagenome. (A) Pathways involved in butyrate production showing the putative genes identified coding for the enzymes involved. Size of pie chart reflects abundance of the gene class; numbers indicate quantity of genes assigned to each taxon. (B) Operon structure of two butyryl-CoA:acetate-CoA transferase (BCD) loci.

We searched the chicken cecal microbiome for BCD sequences, using a protein-sequence-based hidden Markov model, twinned with evidence from genetic context (co-occurrence with adjacent ETF genes). This approach yielded nineteen sequences, mostly from *Bacteroidetes* and *Firmicutes*, but also a single sequence from *Escherichia* spp (Figure 5A). In one *Firmicutes* EGT, there was enough adjacent sequence to reveal genomic context, which included the classic BDH operon structure [61] (Figure 5B). However in *Bacteroidetes* and *Escherichia*, the order of the BCD and ETF genes differed and they were not adjacent to other genes in the butyrate fermentation pathway.

After the formation of butyryl–CoA, butyrate fermentation can proceed either by butyryl-CoA:acetate:CoA transferase or via the enzymes phosphotransbutyrylase and butyrate kinase (Figure 5A). The former pathway is by far the most commonly used by butyrate-producing bacteria isolated from the human gut [61]. We detected only four sequences encoding butyryl-CoA:acetate:CoA transferase in our chicken cecal metagenome. This is in line with results from a recent study of butyrate-producing bacteria cultured from chicken ceca [62], where fewer than half the isolates contained the butyryl-CoA:aceate: CoA transferase gene, leading to speculation that another as yet unidentified acyl transferase might substitute for the canonical butyryl-CoA: acetate CoA transferase in some butyrate fermentation pathways. We found nineteen sequences corresponding to phosphotransbutylase and butyrate kinase, including seven from the *Bacteroidetes*. As *Bacteroidetes* appear to contain an energy conserving BCD/ETF complex and phosphotransbutylase/butyrate kinase genes, we speculate that they might have the potential to produce butyrate. In this context, it is worth noting that, although butyrate production is not generally associated with *Bacteroidetes* grown in culture, butyrate has been shown to be produced by *Bacteroides* spp, in colonized germ free mice [63].

### Hydrogen sinks within the cecal microbiota

One of the goals of metagenomics, and of microbial ecology in general, is to explain why some taxa are so much more abundant than others in a given microbiology community. We speculated that high species abundance might be explained in the chicken cecal microbiome by the need for hydrogen sinks within this ecosystem.

During fermentation, NADH formed through glycolysis needs to be recycled to NAD^+^. This can be achieved through reduction of pyruvate or acetyl CoA to SCFAs. Fermentation to acetate produces the most ATP by substrate level phosphorylation, but this route allows little NADH to be recycled. Hydrogenases provide an alternative route for NADH recycling, but one that leads to the production of molecular hydrogen [47]. Hydrogenases are inhibited by high levels of their own product, so without a sink for hydrogen within an ecosystem, accumulation of hydrogen leads to reduced fermentation and/or less energy-efficient fermentation to substances such as ethanol, butyrate and propionate [19], [64]. The presence of bacteria that can act as a hydrogen sink not only results in a switch to the more productive fermentation to acetate, but also increased production of SCFAs which bring benefits to the host.

**Figure 6:**
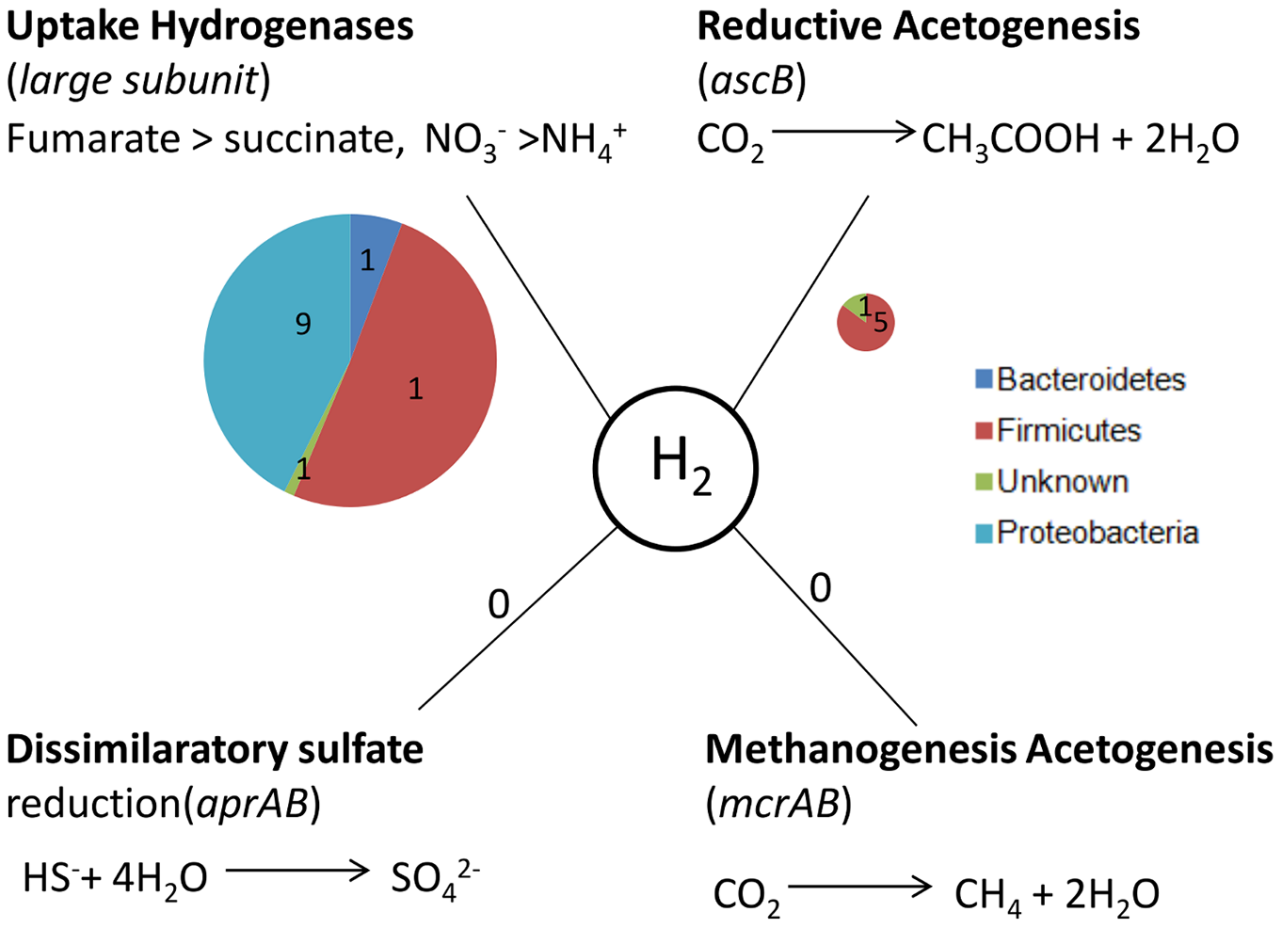
Potential Hydrogen sinks in the Chicken cecal metagenome. The key genes involved in each pathway that could potentially use hydrogen are shown. The size of pie chart reflects abundance of the gene class; numbers indicate quantity of genes assigned to each taxon.

In some enteric microbiotas, methanogens act as major consumers of hydrogen. However, in our dataset, we found no evidence of potential methanogens (members of the *Euryarchaeota*), even though they have been reported in chicken cecal samples in other studies [65]. Sulfate reducers are also potential hydrogen consumer, but we found no evidence of *Desulfovibrio* spp. in our 16S and metagenomic data. Acetogenesis, the reduction of CO_2_ to acetyl-CoA, provides another potential hydrogen sink, but we detected only six acetyl CoA synthase genes, (from the *Ruminococcae* and *Lachnospiraceae*) in our dataset (table S5 and Fig. 6).

Bacterial uptake NiFe hydrogenases link the oxidation of H_2_ to the reduction of anaerobic electron acceptors such as NO_3_^−^, SO_4_^−^, fumarate or CO_2_ [47]. Bacteria that possess these enzymes (such as *Wollinella* and *Campylobacter*) could potentially provide a significant hydrogen sink capable of facilitating acetate production in microbial ecosystems [66], [67]. We found sequences for twelve uptake hydrogenases in our chicken cecal metagenome (Figure 6 and table S5), in several of the most abundant genera in this ecosystem (*Megamonas*, *Helicobacter*, *Campylobacter*). We speculate that the high abundance of these genera in the chicken cecum might, at least in part, be explained by their potential to remove hydrogen, which is likely to benefit not only the other members of the microbial community, but also, indirectly, the host, by improving recovery of energy from food.

## Supporting Information

Figure S1 **Relationship Between Size of OTU and Percentage Identity to Genbank Sequences.** Graph showing relationship between the number of reads in an OTU and its percentage identity to the closest match in the NCBI nr database. Those OTUs showing limited homology still were comprised of many reads hence increasing the validity of the OTU.
(TIF)

Figure S2 **Summary of Previous Studies on the Chicken Metagenome.** Graph showing the relative amounts of different taxa within the chicken ceca from a number of different studies using different techniques (1). J.H.A. Apajalahti and A. Kettunen p. 124–126 in G. Perry, ed. *Avian Gut Function in Health and Disease*,2006. (2). W.E. Holben, K.P. Feris, A. Kettunen and J.H.A Apajalahti Appl. Envron. Microb, 70:2263–2270, 2004. (3). J.R. Lu, U. Idris, B. Harmon, C. Hofacre, J.J. Maurer and M.D. Lee. App. Environ. Microb. 69:6186–6823, 2003. ().G.R. Siragusa and M.G. Wise J. Appl. Microb. 102:1138–1149, 2007. (5). M.D. Cressman, Z. Yu, M.C. Nelson, S.J. Moeller, M.S. Liburn and H.N. Zerby. Appl Environ Microbiol 76:6572–6582 2010. (6). J.L. Danzeisen, H.B. Kim, R.E. Isaacson, Z.J. Tu and T.J. Johnson. Plos One 6:e27949. 2011 (7). This study.
(TIF)

Figure S3 **Taxonomic Assignment of Metagenomic data.** (A) The percentage of each EGT that could be assigned to a given taxonomic level. EGTs were assigned taxonomy based on the protein coding sequences they contained (see methods). (B) Comparison of phylogenetic assignment from pyrosequenced 16S amplicons and EGTs. (i) 260 000 110 bp reads assigned by MEGAN using the Least Common Ancestor algorithm. (ii) Taxonomic assignment of protein sequences from the assembled metagenomic data, abundance corrected by coverage. (iii) Taxonomic distribution of the genomes, abundance corrected by coverage and size of genome. (iv). Taxonomy of 16S, assigned using the rdp database project taxonomy.
(TIF)

Figure S4 **Organization of the Metagenomic Data.** (A) Flow diagram of the procedures used to analyse the metagenomic data. Grey boxes show the programs that were used for each step (B) Database scheama showing the structure of the MySQL database holding the metagenomic information.
(PDF)

Figure S5 **Comparison of OTU pipelines.** (A) Rareaction curves for each OTU picking method. The four reps for each chicken were merged (i) Mothur (ii) Custom (iii) Uparse. (B) PCA plots showing differences in OTU composition of each chicken (colour) when analysed by different pipelines (circle Uparse, triangle custom method and square Mothur). OTUs were merged according to taxonomic assignment by the RDP classifier at the family level. The axes represent the two principal components which are responsible for the most variation in the samples.
(TIF)

Table S1 **Summary of the reads obtained for each of the 10 chickens.**
(DOCX)

Table S2 **Broad Taxonomic assignment of a subset of reads from the chicken metagenome.**
(DOCX)

Table S3 **Genes which contain both GH motifs and carbohydrate binding motifs (CBMs) or dockerin motifs.**
(DOCX)

Table S4 **Predicted acetate kinase/phosphotransferase loci present in the chicken metagenome.**
(DOCX)

Table S5 **Predicted Acetyl CoA synthase genes and the large subunit of uptake hydrogenases present in the Chicken metagenome.**
(DOCX)

Table S6 **The assignment of putative NSP degrading enzymes based on SEED/KEGG annotation and GH domains.**
(DOCX)

Text S1 **Comparisons of different techniques to cluster reads into OTUs from raw reads.**
(DOCX)

Data S1 **List of all OTUs showing assigned taxonomy and their relative abundance in each sample.**
(XLSX)

Data S2 **Table of antibiotic resistance genes present in the metagenome.**
(XLSX)
