# Supplementary figures and images for: Extensive Microbial and Functional Diversity within the Chicken Cecal Microbiome
Source: PLoS One. 2014 Mar 21;9(3):e91941. doi: 10.1371/journal.pone.0091941 (PMC3962364; doi:10.1371/journal.pone.0091941)

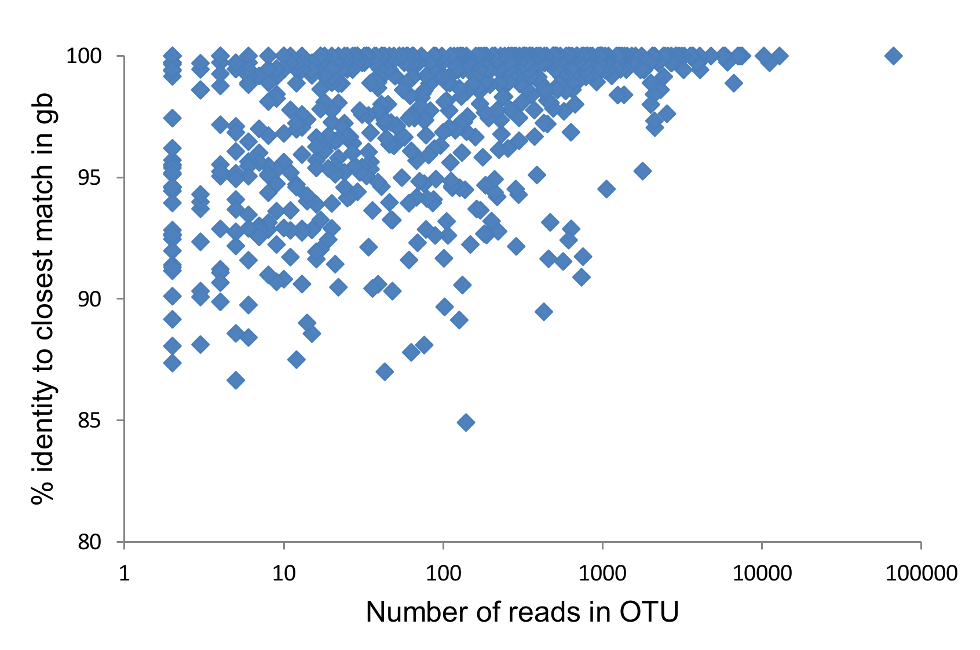

Supplement: Figure S1 — Relationship Between Size of OTU and Percentage Identity to Genbank Sequences. Graph showing relationship between the number of reads in an OTU and its percentage identity to the closest match in the NCBI nr database. Those OTUs showing limited homology still were comprised of many reads hence increasing the validity of the OTU. (TIF) [file pone.0091941.s001.tif]

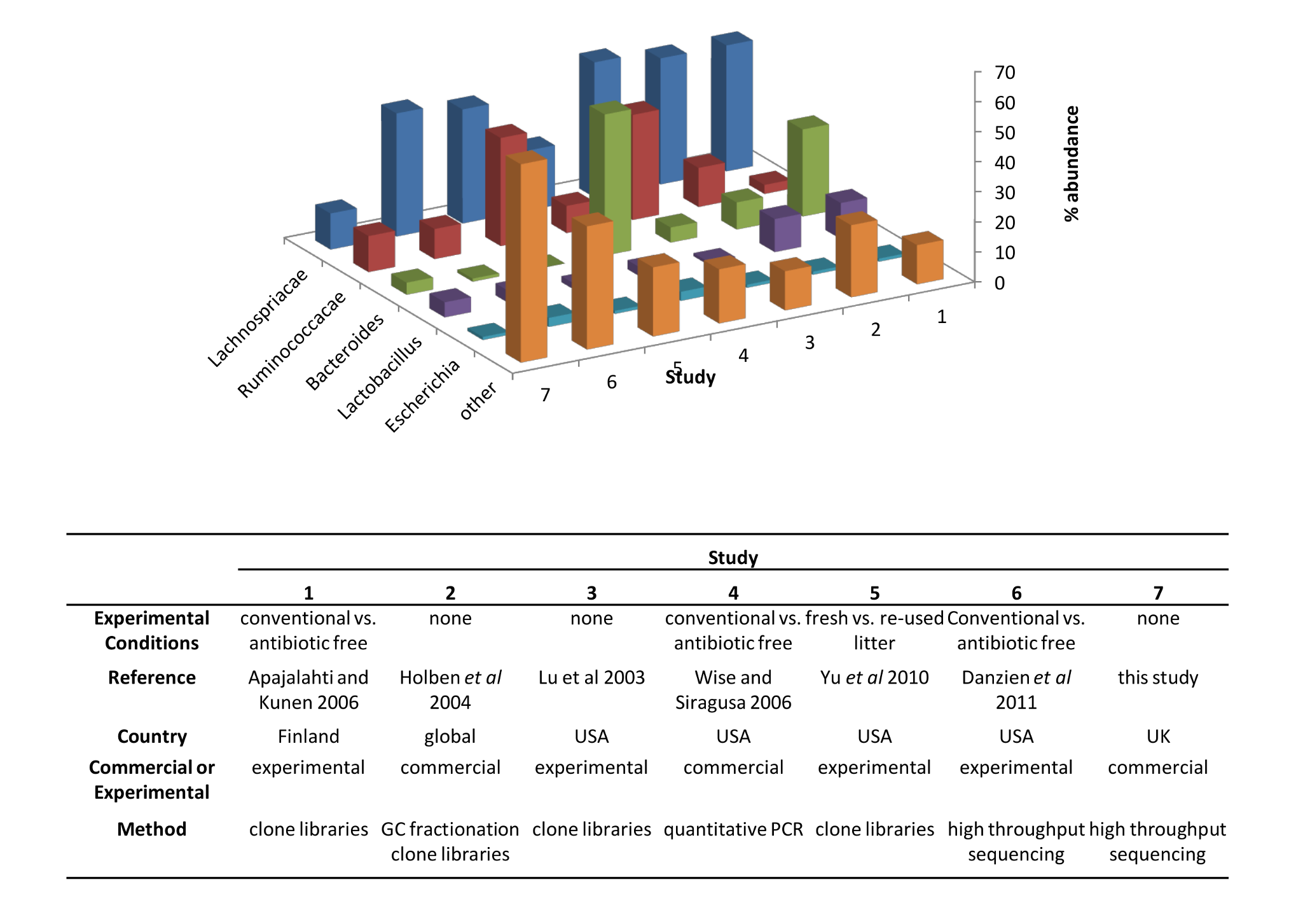

Supplement: Figure S2 — Summary of Previous Studies on the Chicken Metagenome. Graph showing the relative amounts of different taxa within the chicken ceca from a number of different studies using different techniques (1). J.H.A. Apajalahti and A. Kettunen p. 124–126 in G. Perry, ed. Avian Gut Function in Health and Disease,2006. (2). W.E. Holben, K.P. Feris, A. Kettunen and J.H.A Apajalahti Appl. Envron. Microb, 70:2263–2270, 2004. (3). J.R. Lu, U. Idris, B. Harmon, C. Hofacre, J.J. Maurer and M.D. Lee. App. Environ. Microb. 69:6186–6823, 2003. ().G.R. Siragusa and M.G. Wise J. Appl. Microb. 102:1138–1149, 2007. (5). M.D. Cressman, Z. Yu, M.C. Nelson, S.J. Moeller, M.S. Liburn and H.N. Zerby. Appl Environ Microbiol 76:6572–6582 2010. (6). J.L. Danzeisen, H.B. Kim, R.E. Isaacson, Z.J. Tu and T.J. Johnson. Plos One 6:e27949. 2011 (7). This study. (TIF) [file pone.0091941.s002.tif]

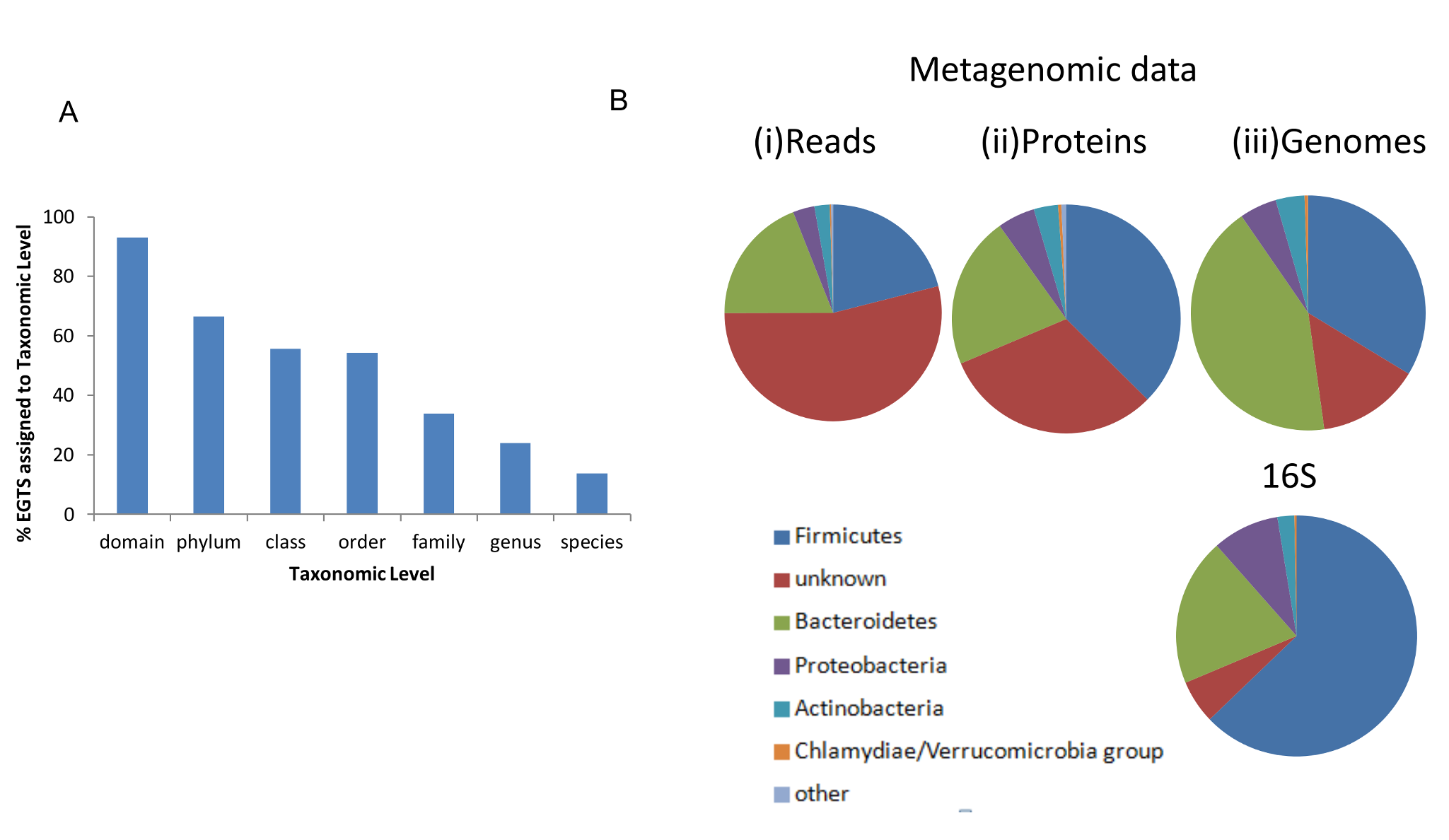

Supplement: Figure S3 — Taxonomic Assignment of Metagenomic data. (A) The percentage of each EGT that could be assigned to a given taxonomic level. EGTs were assigned taxonomy based on the protein coding sequences they contained (see methods). (B) Comparison of phylogenetic assignment from pyrosequenced 16S amplicons and EGTs. (i) 260 000 110 bp reads assigned by MEGAN using the Least Common Ancestor algorithm. (ii) Taxonomic assignment of protein sequences from the assembled metagenomic data, abundance corrected by coverage. (iii) Taxonomic distribution of the genomes, abundance corrected by coverage and size of genome. (iv). Taxonomy of 16S, assigned using the rdp database project taxonomy. (TIF) [file pone.0091941.s003.tif]

A

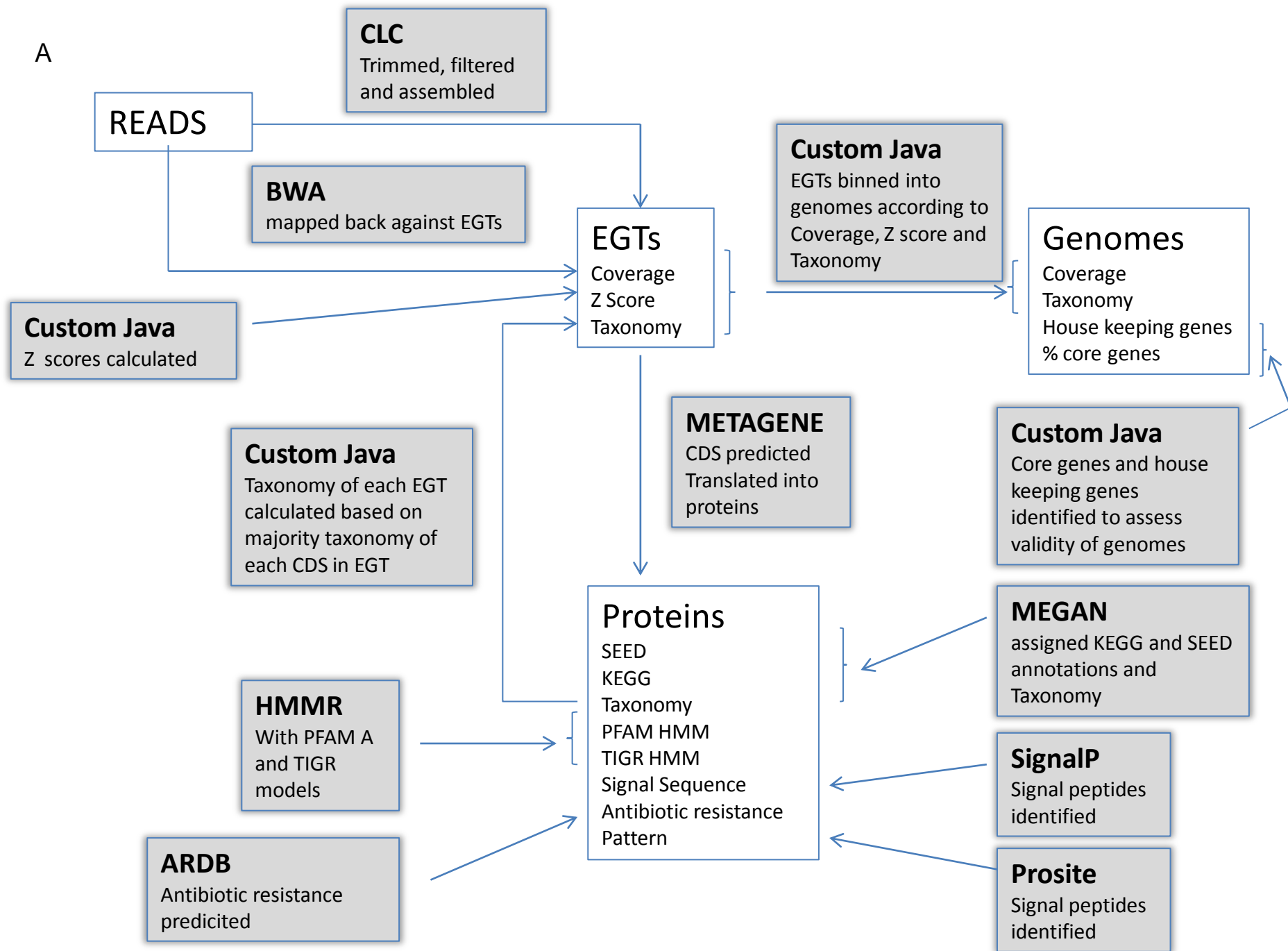

B

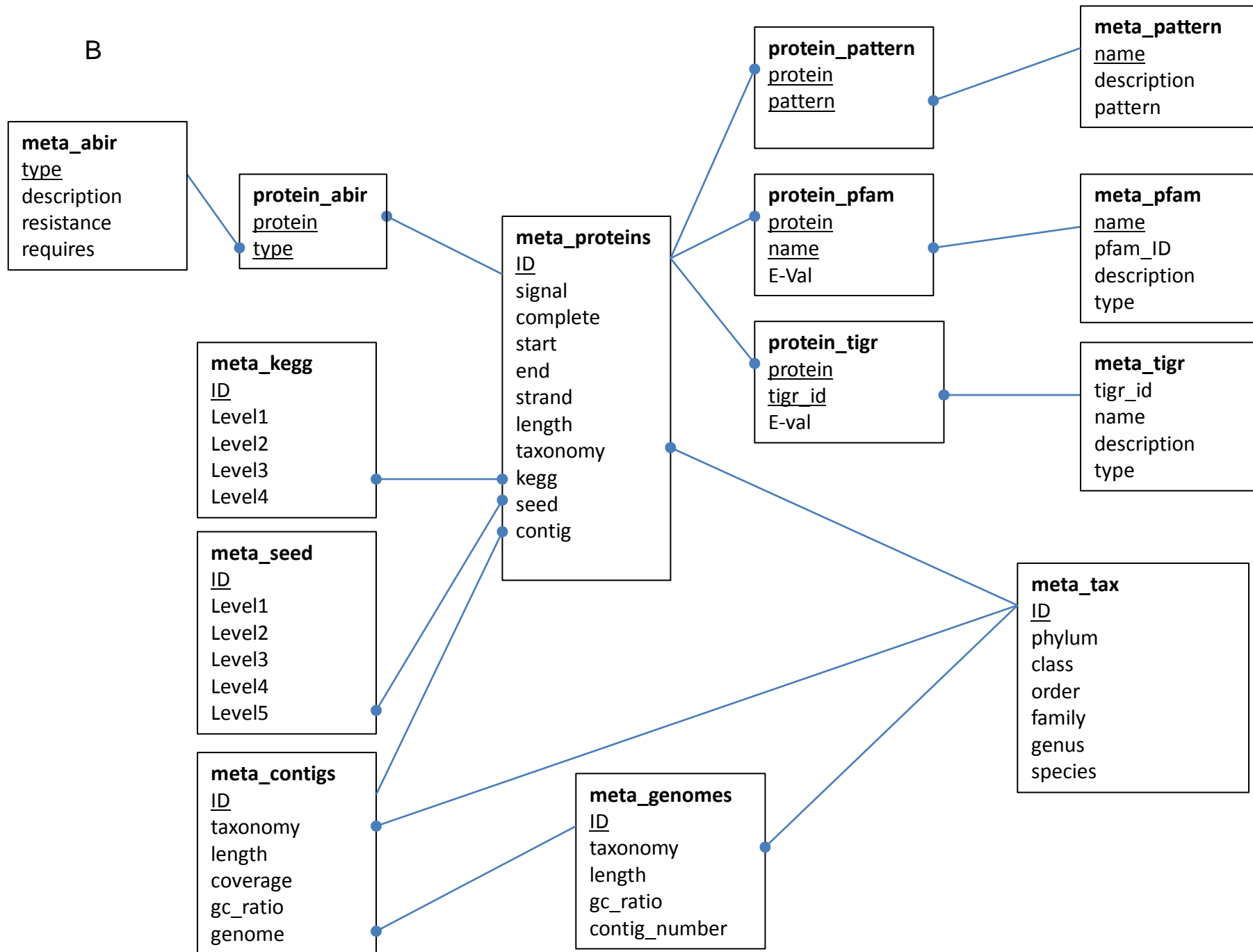

Supplement: Figure S4 — Organization of the Metagenomic Data. (A) Flow diagram of the procedures used to analyse the metagenomic data. Grey boxes show the programs that were used for each step (B) Database scheama showing the structure of the MySQL database holding the metagenomic information. (PDF) [file pone.0091941.s004.pdf]

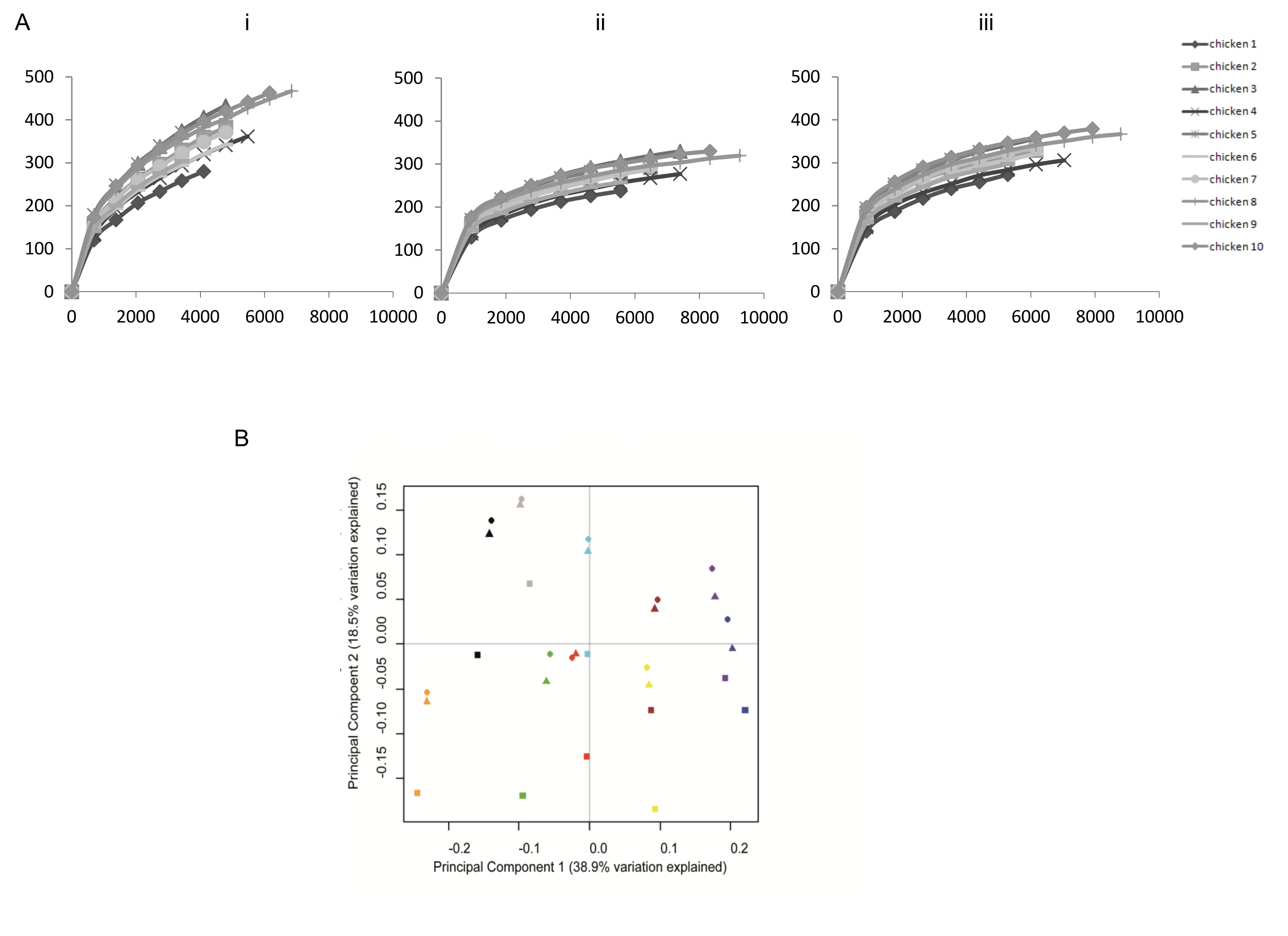

Supplement: Figure S5 — Comparison of OTU pipelines. (A) Rareaction curves for each OTU picking method. The four reps for each chicken were merged (i) Mothur (ii) Custom (iii) Uparse. (B) PCA plots showing differences in OTU composition of each chicken (colour) when analysed by different pipelines (circle Uparse, triangle custom method and square Mothur). OTUs were merged according to taxonomic assignment by the RDP classifier at the family level. The axes represent the two principal components which are responsible for the most variation in the samples. (TIF) [file pone.0091941.s005.tif]
