# Supplementary material for: Extensive Microbial and Functional Diversity within the Chicken Cecal Microbiome
Source: PLoS One. 2014 Mar 21;9(3):e91941. doi: 10.1371/journal.pone.0091941 (PMC3962364; doi:10.1371/journal.pone.0091941)
Supplement: Table S1 — Summary of the reads obtained for each of the 10 chickens. (DOCX) [file pone.0091941.s006.docx]

**Table S1** Summary of the reads obtained for each of the 10 chickens

| Chicken | Ceca | rep | Number of Reads | Number of OTUs^a^ | Simpsons  Index^b^ | Shannon  Index^b^ |
| --- | --- | --- | --- | --- | --- | --- |
| 1 | A | 1 | 25693 | 412 | 0.93 | 5.73 |
| 1 | A | 2 | 7176 | 297 | 0.89 | 5.12 |
| 1 | B | 1 | 6086 | 261 | 0.85 | 4.73 |
| 1 | B | 2 | 8017 | 285 | 0.83 | 4.57 |
| 2 | A | 1 | 14971 | 404 | 0.98 | 6.74 |
| 2 | A | 2 | 10004 | 358 | 0.97 | 6.39 |
| 2 | B | 1 | 9124 | 353 | 0.92 | 5.84 |
| 2 | B | 2 | 7365 | 336 | 0.93 | 5.95 |
| 3 | A | 1 | 7812 | 379 | 0.94 | 6.15 |
| 3 | A | 2 | 9944 | 401 | 0.91 | 5.80 |
| 3 | B | 1 | 9214 | 373 | 0.96 | 6.42 |
| 3 | B | 2 | 7400 | 368 | 0.96 | 6.47 |
| 4 | A | 1 | 8128 | 323 | 0.92 | 5.48 |
| 4 | A | 2 | 8171 | 307 | 0.92 | 5.49 |
| 4 | B | 1 | 8638 | 305 | 0.88 | 5.00 |
| 4 | B | 2 | 8754 | 331 | 0.94 | 5.88 |
| 5 | A | 1 | 5451 | 343 | 0.98 | 6.88 |
| 5 | A | 2 | 11767 | 396 | 0.98 | 6.85 |
| 5 | B | 1 | 7120 | 361 | 0.98 | 6.91 |
| 5 | B | 2 | 15927 | 414 | 0.98 | 6.92 |
| 6 | A | 1 | 7723 | 340 | 0.97 | 6.24 |
| 6 | A | 2 | 14066 | 383 | 0.96 | 6.08 |
| 6 | B | 1 | 16616 | 394 | 0.97 | 6.43 |
| 6 | B | 2 | 10211 | 362 | 0.97 | 6.44 |
| 7 | A | 1 | 7272 | 352 | 0.92 | 5.76 |
| 7 | A | 2 | 12086 | 390 | 0.93 | 5.85 |
| 7 | B | 1 | 7000 | 331 | 0.88 | 5.40 |
| 7 | B | 2 | 8194 | 355 | 0.91 | 5.77 |
| 8 | A | 1 | 11813 | 373 | 0.98 | 6.73 |
| 8 | A | 2 | 20121 | 429 | 0.98 | 6.86 |
| 8 | B | 1 | 9738 | 378 | 0.98 | 6.57 |
| 8 | B | 2 | 13016 | 387 | 0.98 | 6.69 |
| 9 | A | 1 | 13534 | 339 | 0.85 | 5.23 |
| 9 | A | 2 | 5592 | 319 | 0.93 | 6.05 |
| 9 | B | 1 | 9289 | 347 | 0.91 | 5.74 |
| 9 | B | 2 | 9658 | 329 | 0.88 | 5.43 |
| 10 | A | 1 | 9474 | 391 | 0.98 | 6.85 |
| 10 | A | 2 | 10603 | 403 | 0.98 | 6.83 |
| 10 | B | 1 | 8720 | 378 | 0.94 | 6.23 |
| 10 | B | 2 | 12475 | 405 | 0.97 | 6.62 |

_a_Operational Taxonomic Units (OTUs) were calculated at the 97 % identity level ^b^ The Shannon and Simpsons diversity indices were calculated using QIIME
