# Supplementary material for: Extensive Microbial and Functional Diversity within the Chicken Cecal Microbiome
Source: PLoS One. 2014 Mar 21;9(3):e91941. doi: 10.1371/journal.pone.0091941 (PMC3962364; doi:10.1371/journal.pone.0091941)
Supplement: Table S2 — Broad Taxonomic assignment of a subset of reads from the chicken metagenome. (DOCX) [file pone.0091941.s007.docx]

**Table S2** Broad Taxonomic assignment of a subset of reads from the chicken metagenome

| Taxon^a^ | Number of Reads |
| --- | --- |
| Bacteria | 119921 |
| Archea | 49 |
| *Methanococcus maripaludis* | 9 |
| *Methanocorpusculum labreanum* | 7 |
|  |  |
| Eukaryota | 2542 |
| *Galus Galus* | 847 |
| *Magnaporthe orazae* | 54 |
| *Chlamydomanas reinhardtii* | 29 |
|  |  |
| Viruses | 110 |
| *Caudovirals (*phages) | 95 |
| Fowl Adenovirus | 7 |
|  | 21 |
| Not assigned | 39747 |
| No hit | 96205 |

^a^Taxonomic information was obtained by blastx of raw reads against the NCBI nr database and the resulting output analysed with MEGAN to assign putative taxonomy. The data shows the results of a subset of 260 000 reads.
