# Supplementary material for: Extensive Microbial and Functional Diversity within the Chicken Cecal Microbiome
Source: PLoS One. 2014 Mar 21;9(3):e91941. doi: 10.1371/journal.pone.0091941 (PMC3962364; doi:10.1371/journal.pone.0091941)
Supplement: Table S3 — Genes which contain both GH motifs and carbohydrate binding motifs (CBMs) or dockerin motifs. (DOCX) [file pone.0091941.s008.docx]

| **GH** | **Substrate^a^** | **CBM** | **Target^a^** | **total coverage** | **taxonomy** | **number** |
| --- | --- | --- | --- | --- | --- | --- |
| GH110 | α-galactosidase, α-1,3-galactosidase | Dockerin_1 | cellulosome? | 21 | Actinobacteria | 1 |
| GH16 | endo-1,4-β-galactosidase, endo-1,3-β-glucanase, endo-1,3(4)-β-glucanase, licheninase, β-agarase, other | CBM_4_9 | xylan, β-1,3-glucan, β-1,3-1,4-glucan, β-1,6-glucan and amorphous cellulose binding domain | 73 | Firmicutes | 1 |
| GH26 | β-mannanase, β-1,3-xylanase | CBM_6 | amorphous cellulose and xylan-binding domain | 8 | Firmicutes | 1 |
| GH2 | β-galactosidase, β-glucuronidase, β-mannosidase,others | CBM_11 | glucan binding domain | 13 | Chlamydiae/Verrucomicrobia group | 1 |
| GH31 | α-glucosidase, α-1,3-glucosidase, α-xylosidase | CBM_6 | amorphous cellulose and xylan-binding domain | 29 | Firmicutes,unknown | 3 |
| GH32 | levanase, invertase, others | CBM_38 | inulin binding domain | 51 | Firmicutes | 1 |
| GH42 | β-galactosidase | CBM_11 | glucan binding domain | 13 | Chlamydiae/Verrucomicrobia group | 1 |
| GH42 | β-galactosidase | CBM_4_9 | xylan, β-1,3-glucan, β-1,3-1,4-glucan, β-1,6-glucan and amorphous cellulose binding domain | 24 | Chlamydiae/Verrucomicrobia group,unknown | 2 |
| GH43 | β-xylosidase, β-1,3-xylosidase, α-Larabinofuranosidase,arabinanase, xylanase, galactan,1,3-β-galactosidase | CBM_36 | xylans and xylooligosaccharides binding domain | 43 | Firmicutes,Bacteroides,unknown | 3 |
| GH43 | β-xylosidase, β-1,3-xylosidase, α-Larabinofuranosidase,arabinanase, xylanase, galactan,1,3-β-galactosidase | CBM_38 | inulin binding domain | 51 | Firmicutes | 1 |
| GH43 | β-xylosidase, β-1,3-xylosidase, α-Larabinofuranosidase,arabinanase, xylanase, galactan,1,3-β-galactosidase | CBM_4_9 | xylan, β-1,3-glucan, β-1,3-1,4-glucan, β-1,6-glucan and amorphous cellulose binding domain | 8 | Firmicutes | 1 |
| GH43 | β-xylosidase, β-1,3-xylosidase, α-Larabinofuranosidase,arabinanase, xylanase, galactan,1,3-β-galactosidase | CBM_6 | amorphous cellulose and xylan-binding domain | 117 | Firmicutes/unknown/Bacterodetes | 6 |
| GH51 | α-L-arabinofuranosidase, endoglucanase | CBM_4_9 | xylan, β-1,3-glucan, β-1,3-1,4-glucan, β-1,6-glucan and amorphous cellulose binding domain | 32 | Bacteroidetes | 1 |
| GH77 | amylomaltase or 4-α-glucanotransferase | CBM_20 | starch binding domain | 113 | Bacteroidetes | 4 |
| GH92 | α-1,2-mannosidase | CBM_6 | amorphous cellulose and xylan-binding domain | 11 | Firmicutes | 1 |
| GH94 | cellobiose phosphorylase, cellodextrin phosphorylase,chitobiose phosphorylase, cyclic β-1,2-glucan,synthase | CBM_X | unknown | 420 | Firmicutes,unknown | 24 |
| GH95 | α-1,2-L-fucosidase, α-L-fucosidase | Dockerin_1 | cellulosome? | 21 | Firmicutes | 1 |

**Table S3**. Genes which contain both GH motifs and carbohydrate binding motifs (CBMs) or dockerin motifs

^a^ information about the potential substrate and target of the cellulose binding domain (CBM) were taken from the Cazy website (http://www.cazy.org/)
