# Supplementary material for: Extensive Microbial and Functional Diversity within the Chicken Cecal Microbiome
Source: PLoS One. 2014 Mar 21;9(3):e91941. doi: 10.1371/journal.pone.0091941 (PMC3962364; doi:10.1371/journal.pone.0091941)
Supplement: Table S4 — Predicted acetate kinase/phosphotransferase loci present in the chicken metagenome. (DOCX) [file pone.0091941.s009.docx]

|  | Predicted Taxonomy^a^ | | | | |  |
| --- | --- | --- | --- | --- | --- | --- |
| Contig | Phylum | Class | Order | Family | Coverage | |
| c97896 | Proteobacteria | Epsilonproteobacteria | Campylobacterales | Helicobacteraceae | 83 | |
| c98240 | Bacteroidetes | unknown | unknown | unknown | 43 | |
| c105091 | Bacteroidetes | Bacteroidia | Bacteroidales | Rikenellaceae | 14 | |
| c109173 | Firmicutes | Clostridia | Clostridiales | unknown | 11 | |
| c130567 | Firmicutes | Clostridia | Clostridiales | unknown | 10 | |
| c362621 | Proteobacteria | Epsilonproteobacteria | Campylobacterales | Campylobacteraceae | 39 | |
| c363024 | Bacteroidetes | Bacteroidia | Bacteroidales | Rikenellaceae | 37 | |
| c363279 | unknown | unknown | unknown | unknown | 27 | |
| c363324 | Actinobacteria | Actinobacteria | Coriobacteriales | Coriobacteriaceae | 50 | |
| c363363 | Firmicutes | Clostridia | Clostridiales | unknown | 73 | |
| c364002 | Firmicutes | Clostridia | Clostridiales | Lachnospiraceae | 16 | |
| c364270 | Proteobacteria | Gammaproteobacteria | Enterobacteriales | Enterobacteriaceae | 21 | |
| c364329 | Bacteroidetes | Bacteroidia | Bacteroidales | Bacteroidaceae | 15 | |
| c364809 | Firmicutes | Clostridia | Clostridiales | Ruminococcaceae | 16 | |
| c364867 | unknown | unknown | unknown | unknown | 31 | |
| c365055 | Bacteroidetes | Bacteroidia | Bacteroidales | Bacteroidaceae | 23 | |
| c366133 | unknown | unknown | unknown | unknown | 11 | |
| c367484 | unknown | unknown | unknown | unknown | 15 | |
| c367862 | Bacteroidetes | Bacteroidia | Bacteroidales | Bacteroidaceae | 33 | |
| c369506 | Firmicutes | Clostridia | Clostridiales | Lachnospiraceae | 16 | |
| c374314 | unknown | unknown | unknown | unknown | 28 | |
| c374335 | Firmicutes | Clostridia | Clostridiales | Lachnospiraceae | 16 | |
| c374350 | unknown | unknown | unknown | unknown | 11 | |
| c401093 | unknown | unknown | unknown | unknown | 13 | |
| c433017 | unknown | unknown | unknown | unknown | 3 | |
| c487669 | unknown | unknown | unknown | unknown | 60 | |
| c498731 | Firmicutes | Clostridia | Clostridiales | unknown | 8 | |
| c599550 | Bacteroidetes | Bacteroidia | Bacteroidales | Rikenellaceae | 982 | |
| c599702 | Bacteroidetes | unknown | unknown | unknown | 55 | |
| c600052 | Firmicutes | Clostridia | Clostridiales | Ruminococcaceae | 48 | |
| c600279 | Bacteroidetes | Bacteroidia | Bacteroidales | unknown | 27 | |
| c601906 | unknown | unknown | unknown | unknown | 11 | |

**Table S4** Predicted acetate kinase/phosphotransferase loci present in the chicken metagenome

Loci shown are where the acetate kinase (TIGR0016) and acetate phosphotransferase (TIGR00651) genes are located adjacent to each other ^a^Taxonomy based on the taxonomic assignment of both genes using the LCA algorithm implemented by MEGAN.
