# Supplementary material for: Extensive Microbial and Functional Diversity within the Chicken Cecal Microbiome
Source: PLoS One. 2014 Mar 21;9(3):e91941. doi: 10.1371/journal.pone.0091941 (PMC3962364; doi:10.1371/journal.pone.0091941)
Supplement: Table S5 — Predicted Acetyl CoA synthase genes and the large subunit of uptake hydrogenases present in the Chicken metagenome. (DOCX) [file pone.0091941.s010.docx]

|  |  | Predicted Taxonomy^a^ | | | | |  |  |
| --- | --- | --- | --- | --- | --- | --- | --- | --- |
|  | id | Phylum | Class | Order | Family | Genus | Coverage | Contig |
| acetyl CoA synthase | 138182 | Firmicutes | unknown | unknown | unknown | unknown | 11 | c127943 |
|  | 415785 | Firmicutes | unknown | unknown | unknown | unknown | 9 | c372233 |
|  | 426338 | Firmicutes | unknown | unknown | unknown | unknown | 12 | c375276 |
|  | 552410 | Firmicutes | unknown | unknown | unknown | unknown | 12 | c489336 |
|  | 595473 | Firmicutes | unknown | unknown | unknown | unknown | 7 | c529883 |
|  | 659793 | unknown | unknown | unknown | unknown | unknown | 19 | c603330 |
| Uptake hydrogenases  (large subunit) | 365484 | Bacteroidetes | unknown | unknown | unknown | unknown | 41 | c362549 |
|  | 546202 | Firmicutes | Negativicutes | Selenomonadales | Veillonellaceae | Megamonas | 361 | c487620 |
|  | 84680 | Proteobacteria | Epsilonproteobacteria | Campylobacterales | Helicobacteraceae | Helicobacter | 94 | c97918 |
|  | 102231 | Proteobacteria | Gammaproteobacteria | Enterobacteriales | Enterobacteriaceae | unknown | 19 | c103880 |
|  | 274705 | Proteobacteria | Deltaproteobacteria | Desulfovibrionales | Desulfovibrionaceae | unknown | 4 | c263446 |
|  | 365743 | Proteobacteria | Epsilonproteobacteria | Campylobacterales | Campylobacteraceae | Campylobacter | 43 | c362553 |
|  | 385297 | Proteobacteria | Betaproteobacteria | Burkholderiales | unknown | unknown | 12 | c363929 |
|  | 460216 | Proteobacteria | Gammaproteobacteria | Enterobacteriales | Enterobacteriaceae | unknown | 22 | c393467 |
|  | 477428 | Proteobacteria | Gammaproteobacteria | Enterobacteriales | Enterobacteriaceae | unknown | 19 | c405140 |
|  | 478540 | Proteobacteria | Gammaproteobacteria | Enterobacteriales | Enterobacteriaceae | unknown | 47 | c406023 |
|  | 485825 | Proteobacteria | Gammaproteobacteria | Enterobacteriales | Enterobacteriaceae | unknown | 44 | c411498 |
|  | 12266 | unknown | unknown | unknown | unknown | unknown | 8 | c13070 |

**Table S5**. Predicted Acetyl CoA synthase genes and the large subunit of uptake hydrogenases present in the Chicken metagenome

The acetyl CoA synthase and uptake dehydrogenases were predicted based on specific patterns (see methods) ^a^Taxonomy based on the taxonomic assignment of both genes using the LCA algorithm implemented by MEGAN.
