# Supplementary material for: Extensive Microbial and Functional Diversity within the Chicken Cecal Microbiome
Source: PLoS One. 2014 Mar 21;9(3):e91941. doi: 10.1371/journal.pone.0091941 (PMC3962364; doi:10.1371/journal.pone.0091941)
Supplement: Table S6 — The assignment of putative NSP degrading enzymes based on SEED/KEGG annotation and GH domains. (DOCX) [file pone.0091941.s011.docx]

| **EC Number in SEED or KEGG anotation** | **Name** | **Description** | **GH domains** |
| --- | --- | --- | --- |
| **xylanases** |  |  |  |
| 3.2.1.8 | endo 1-4 β xylanase | endohydrolysis of 1,4 β D xylosidic linkages in xylans to release oligomers of xylan . The final product in Clostridium thermocellum is the disaccharide xylobiose | GH5, GH8, GH10, GH11, GH43, CBM_4_9 |
| 3.2.1.37 | Xylan 1,4-beta-xylosidase. | Hydrolysis of (1->4)-beta-D-xylans, to remove successive D-xylose residues from the non-reducing termini | GH1, GH3, GH30, GH39, GH43, GH52, GH54, GH116, GH120 and glycol_hydro_cc, cellulose like and CBM_4_9 |
| 3.2.1.55 | Alpha-N-arabinofuranosidase | Hydrolysis of terminal non-reducing alpha-L-arabinofuranoside residues in alpha-L-arabinosides | GH3, GH43, GH51, GH54, GH62, Alpha-L-AF_C, CBM_4_9 |
| **cellulases** |  |  |  |
| 3.2.1.91 | Cellulose 1,4-beta-cellobiosidase (non-reducing end). | Hydrolysis of (1->4)-beta-D-glucosidic linkages in cellulose and cellotetraose, releasing cellobiose from the non-reducing ends of the chains | GH5, GH6, GH9 |
| 3.2.1.8 | Cellulase | Endohydrolysis of (1->4)-beta-D-glucosidic linkages in cellulose, lichenin and cereal beta-D-glucans | GH5, GH6, GH7, GH8, GH9, GH12, GH44, GH45, GH48, GH51, CelD_N |
| 3.2.1.21 | Beta-glucosidase | Hydrolysis of terminal, non-reducing beta-D-glucosyl residues with release of beta-D-glucose | GH1, GH3, GH9, GH30, GH116 |
| **lichinases** |  |  |  |
| 3.2.1.73 | Licheninase | Hydrolysis of (1->4)-beta-D-glucosidic linkages in beta-D-glucans containing (1->3)- and (1->4)-bonds | GH5, GH7, GH8, GH12, GH16, GH17, CBM_4_9 |
| 3.2.1.58 | Glucan 1,3-beta-glucosidase | Successive hydrolysis of beta-D-glucose units from the non-reducing ends of (1->3)-beta-D-glucans, releasing alpha-glucose | GH3, GH5, GH17, GH55 |

**Table S5**. The assignment of putative NSP degrading enzymes based on SEED/KEGG annotation and GH domains
