# Supplementary material for: Extensive Microbial and Functional Diversity within the Chicken Cecal Microbiome
Source: PLoS One. 2014 Mar 21;9(3):e91941. doi: 10.1371/journal.pone.0091941 (PMC3962364; doi:10.1371/journal.pone.0091941)
Supplement: Text S1 — Comparisons of different techniques to cluster reads into OTUs from raw reads. (DOCX) [file pone.0091941.s012.docx]

**Supplemental Methods – Pipelines for generating OTUs**

There are many pipelines for processing high-throughput 16S reads, most of which cluster the reads at the 97% identity level. Which pipelines are best depends on the nature of the data and there are contradictory findings in the literature ([Cai and Sun, 2011](#_ENREF_1); [Quince et al., 2011](#_ENREF_7); [Schloss and Westcott, 2011](#_ENREF_8); [Edgar, 2013](#_ENREF_5)). Usually all methods perform well on mock data consisting of a mixture of DNA from a few known species, present at equal concentrations. Their effectiveness against real data, however, is hard to ascertain since the composition of real samples is obviously unknown. With the data that we obtained, conventional methods such as the Mothur pipeline([Schloss et al., 2009](#_ENREF_9)), which include AmpliconNoise ([Quince et al., 2011](#_ENREF_7)) step, were attempted. However these tended to produce thousands of OTUs, suggesting that sequencing errors and artefacts such as chimers were not being removed. Furthermore, for a given taxonomic classification there tended to be one OTU containing the majority of reads and showing a close match to sequences from genbank (>99%) and many other OTUs which contained very few reads that showed less than 97% identity to any sequence in genbank, thus suggesting that these smaller OTUs were due to errors and artefacts of the main OTU.

A custom method was therefore developed whereby sequences were clustered at the 97% identity level by Usearch ([Edgar, 2010](#_ENREF_4)) . Reads in each cluster were then aligned using Muscle ([Edgar, 2004](#_ENREF_3)) and the majority base at each position of the alignment was then used to form the consensus. The consensus sequences were then re-clustered and the process repeated until the number of clusters remained the same. Putative OTU sequences were subject to chimera removal by Perseus ([Quince et al., 2011](#_ENREF_7))and then UChime ([Edgar et al., 2011](#_ENREF_6)). Using more than one chimera removal method proved more robust and working on the OTU consensus sequences rather than the individual reads was far less computationally expensive. Next, SSU-align, which uses Infernal ([Eddy et al., 2009](#_ENREF_2)), was used to align the putative sequences to a 16S model. The resulting alignment (.stk) file was parsed looking for insertions or deletions in the non-masked regions where the confidence values within a 4 bp window were less than 100%. If a sequence failed to align or had greater than six indels it was discarded.

The data was also analysed using the latest pipeline Uparse ([Edgar, 2013](#_ENREF_5)) and this was compared to the Mothur pipeline and the custom method. The number of OTUs generate was >2000, 699 and 668 for the Mothur, Uparse and custom pipelines respectively. Rarefaction curves (Figure S5A) showed that for the Mothur pipeline, the curve appeared not to be reaching an asymptote. Thus implying the new sequences being discovered at higher sequencing depths were just spurious sequences caused by random errors. The OTU compositions produced by each pipeline were also compared by merging OTUs that showed the same taxonomic classification at the family level. PCA plots were then generated using the jackknifed_beta_diversity script from QIIME, exploiting the Bray Curtis method to compute a similarity matrix. From these results (Figure S5B) the Uparse and custom method produced OTU compositions that were similar and different to those produced by the Mothur pipeline.

**References**

Cai, Y., and Sun, Y. (2011) ESPRIT-Tree: hierarchical clustering analysis of millions of 16S rRNA pyrosequences in quasilinear computational time. *Nucleic Acids Research* **39**: e95.

Eddy, S.R., Nawrocki, E.P., and Kolbe, D.L. (2009) Infernal 1.0: inference of RNA alignments. *Bioinformatics* **25**: 1335-1337.

Edgar, R.C. (2004) MUSCLE: multiple sequence alignment with high accuracy and high throughput. *Nucleic Acids Research* **32**: 1792-1797.

Edgar, R.C. (2010) Search and clustering orders of magnitude faster than BLAST. *Bioinformatics* **26**: 2460-2461.

Edgar, R.C. (2013) UPARSE: highly accurate OTU sequences from microbial amplicon reads. *Nature Methods* **10**: 996-998.

Edgar, R.C., Haas, B.J., Clemente, J.C., Quince, C., and Knight, R. (2011) UCHIME improves sensitivity and speed of chimera detection. *Bioinformatics* **27**: 2194-2200.

Quince, C., Lanzen, A., Davenport, R.J., and Turnbaugh, P.J. (2011) Removing Noise From Pyrosequenced Amplicons. *Bmc Bioinformatics* **12**: -.

Schloss, P.D., and Westcott, S.L. (2011) Assessing and improving methods used in operational taxonomic unit-based approaches for 16S rRNA gene sequence analysis. *Appl Environ Microbiol* **77**: 3219-3226.

Schloss, P.D., Westcott, S.L., Ryabin, T., Hall, J.R., Hartmann, M., Hollister, E.B. et al. (2009) Introducing mothur: Open-Source, Platform-Independent, Community-Supported Software for Describing and Comparing Microbial Communities. *Applied and Environmental Microbiology* **75**: 7537-7541.
